# Research Review: Help‐seeking intentions, behaviors, and barriers in college students – a systematic review and meta‐analysis

**DOI:** 10.1111/jcpp.14145

**Published:** 2025-03-12

**Authors:** Ruiying Zhao, Yagmur Amanvermez, Julia Pei, Franchesca Castro‐Ramirez, Charlene Rapsey, Claudia Garcia, David D. Ebert, Josep Maria Haro, Liviu A. Fodor, Oana A. David, Osiris Rankin, Sook Ning Chua, Vania Martínez, Ronny Bruffaerts, Ronald C. Kessler, Pim Cuijpers

**Affiliations:** ^1^ Department of Clinical, Neuro and Developmental Psychology Amsterdam Public Health Research Institute, Vrije Universiteit Amsterdam Amsterdam The Netherlands; ^2^ Department of Medical and Clinical Psychology Tilburg University Tilburg The Netherlands; ^3^ Department of Psychiatry Faculty of Medicine, University of British Columbia Vancouver BC Canada; ^4^ Department of Psychology Harvard University Cambridge MA USA; ^5^ Department of Psychological Medicine University of Otago Dunedin New Zealand; ^6^ Department of Sports and Health Sciences Technical University of Munich Munich Germany; ^7^ Research and Development Unit Parc Sanitari Sant Joan de Déu, CIBERSAM, ISCIII Barcelona Spain; ^8^ DATA Lab International Institute for Advanced Studies of Psychotherapy and Applied Mental Health, Babeș‐Bolyai University Cluj‐Napoca Romania; ^9^ Department of Clinical Psychology and Psychotherapy Babeș‐Bolyai University Cluj‐Napoca Romania; ^10^ Relate Mental Health Malaysia Kuala Lumpur Malaysia; ^11^ CEMERA, Facultad de Medicina Universidad de Chile, and Millennium Nucleus to Improve the Mental Health of Adolescents and Youths (Imhay) Santiago Chile; ^12^ Center for Public Health Psychiatry Katholieke Universiteit Leuven Leuven Belgium; ^13^ Department of Health Care Policy Harvard Medical School Boston MA USA

**Keywords:** Help‐seeking, college students, help‐seeking behaviors, help‐seeking intentions, mental health barriers

## Abstract

**Background:**

The prevalence of mental health problems among college students has increased over the past decade. Even when mental health services are available, many students still struggle to access these services. This systematic review and meta‐analysis aimed to identify the rates at which students actively seek or consider using formal help and to determine the main reasons for not seeking help.

**Methods:**

A comprehensive literature search was conducted on PubMed, PsycINFO, and Embase to identify studies on help‐seeking behaviors, intentions, and barriers to help‐seeking among college students with mental health problems. Random effect models were used to calculate the pooled proportions.

**Results:**

Of the 8,919 identified studies, 62 met the inclusion criteria and were included (*n* = 53 on help‐seeking behaviors, *n* = 21 on help‐seeking intentions, and *n* = 14 on treatment barriers). The pooled prevalence of active help‐seeking behaviors was 28% (179,915/435,768 individuals; 95% CI: 23%–33%, *I* ^2^ = 99.6%), and the aggregated prevalence of help‐seeking intentions was 41% (62,456/80161 individuals; 95% CI: 26%–58%, *I* ^2^ = 99.8%). Common barriers reported by students included a preference to address issues on their own, time constraints, insufficient knowledge of accessible resources, and a perceived lack of need for professional help.

**Conclusions:**

The findings highlight the gap between the mental health needs of the students and their actual help‐seeking rates. Although personal barriers are common, systemic or contextual challenges also affect college students' help‐seeking behaviors.

## Introduction

The college years represent a critical stage during which students undergo the transition from late adolescence into emerging adulthood (Arnett, 2000). Most mental disorders have their onset during this period (Kessler, Amminger, Aguilar‐Gaxiola, Alonso, & Lee, 2007), and a considerable number of students enter college with already established mental health conditions (Solmi et al., 2022). According to the findings of the World Health Organization's World Mental Health International College Student (WMH‐ICS) Initiative, 35% of first‐year college students meet criteria for a lifetime mental disorder, whereas 31% of incoming college students experienced at least one common mental disorder in the past year (Auerbach et al., 2018).

Several adverse consequences can occur when mental disorders remain untreated, such as poor academic performance, high rates of study dropout, disrupted relationships with peers and families, and low quality of life (Bruffaerts et al., 2018; Buchanan, 2012; Hunt & Eisenberg, 2010; Hysenbegasi et al., 2005; Kessler, Walters, & Forthofer, 1998). In addition, these problems may progress to more serious mental health problems (Altamura et al., 2010; Altamura, Santini, Salvadori, & Mundo, 2005; Kessler & Price, 1993; Kisely, Scott, Denney, & Simon, 2006). These negative outcomes highlight the importance of access to professional treatment.

Several effective treatments are available for college students with mental disorders (Cuijpers et al., 2016; Harrer, Adam, et al., 2019). In particular, some colleges, especially in high‐income countries, have provided a range of mental health resources, including group therapy, peer‐support programs, individual counseling, and mental health awareness training for faculty and staff (Abrams, 2022; Lisiecka, Chimicz, & Lewicka‐Zelent, 2023; Priestley, Broglia, Hughes, & Spanner, 2022). To improve accessibility, several colleges have increasingly adopted remote service delivery through digital interventions and apps (Taylor et al., 2024). This trend accelerated during the COVID‐19 pandemic, as traditional in‐person formats were restricted, and college students faced worsened mental health conditions due to reduced social contact and increased loneliness (Koelen et al., 2021; Pandya & Lodha, 2022).

However, despite these resources, college students with mental health conditions continue to show both relatively low intentions to seek help and limited help‐seeking behaviors (Abassi, Fekih‐Romdhane, Maktouf, & Moalla, 2021). Studies found that even as students' mental health needs increased during the pandemic, help‐seeking did not correspondingly rise (Lee, Jeong, & Kim, 2021; Yonemoto & Kawashima, 2023). Help‐seeking intentions refer to a person's willingness to seek professional psychological help to alleviate psychological distress (Vogel, Wade, & Hackler, 2007), whereas help‐seeking behaviors are defined as the actual actions and efforts taken to access professional psychological assistance (Cramer, 1999; Eisenberg, Hunt, & Speer, 2012).

The disparity between the high prevalence of mental health problems, affecting one‐third of students (Auerbach et al., 2018), and the limited service use (Osborn, Li, Saunders, & Fonagy, 2022) raises a crucial question about why students do not seek professional help when needed. To better understand the gap between the available professional resources and their limited use, several studies have investigated college students' help‐seeking intentions, behaviors, and barriers encountered when seeking mental health help (Ebert, Franke, et al., 2019; Ebert, Mortier, et al., 2019; Eisenberg et al., 2012; Hunt & Eisenberg, 2010; Vidourek, King, Nabors, & Merianos, 2014).

A wide variety of barriers could play a role in help‐seeking intentions and behaviors, including financial reasons (SAMSA, 2014). However, within the context of higher education, financial cost may not be the predominant factor, especially not in high‐income countries where universities often offer free or affordable services. Attitudinal barriers, such as stigma or the perception that available help is ineffective, have also been cited as reasons for not seeking help. However, these attitudinal barriers may be declining (Apolinário‐Hagen et al., 2020). Students often express a preference for help‐seeking from informal sources such as their friends or families or do not experience a perceived need (Eisenberg, Hunt, Speer, & Zivin, 2011).

Although a recent meta‐analysis explored the use of mental health services among college students, it did not specifically examine the intentions or behaviors of help‐seeking (Osborn et al., 2022). Similarly, two previous systematic reviews examined barriers to mental health help‐seeking among college students, but only listed barriers reported by students in the studies (Gulliver, Griffiths, & Christensen, 2010; Lui, Sagar‐Ouriaghli, & Brown, 2022), without estimating a pooled proportion for each barrier.

In the present study, the aim was to perform an up‐to‐date systematic review and meta‐analysis to explore the prevalence of help‐seeking behavior and help‐seeking intentions among college students with mental health problems. Additionally, this study evaluated the prevalence of self‐reported barriers that prevent college students with mental health issues from seeking help from mental health professionals.

## Methods

### Protocol and registration

This meta‐analysis followed the reporting standards outlined by the updated PRISMA 2020 guidelines (Page et al., 2021). This study was preregistered at the Open Science Framework: https://osf.io/wemzf.

### Information sources and eligibility criteria

A systematic search was performed in three databases (PubMed, PsycINFO, and Embase) using predefined search strings (the full search string can be found in Appendix S1). The comprehensive databasess search was conducted on 23 June 2021 and updated on 16 December 2023.

The present meta‐analysis included empirical studies reporting the prevalence of help‐seeking behaviors and/or intentions among college students experiencing mental health conditions (Table 1). Barrier data were extracted from selected studies if reasons for not seeking help were provided. Formal sources of help‐seeking included qualified mental health professionals within or outside the university and general practitioners. The term “mental health conditions” was broadly defined to include students meeting DSM/ICD criteria for a disorder (current, 12‐month, or lifetime) or showing high distress on validated tools (e.g., PHQ‐9, GAD‐7). Help‐seeking intentions were broadly defined as any plans to communicate mental health problems with a mental health professional for relief (White, Clough, & Casey, 2018). Therefore, it was conceptualized to include any future actions related to the use of mental health services, such as the likelihood of using these services and the perceived need for them. Inclusion was limited to studies published in peer‐reviewed sources and excluded those with an experimental design, providing only qualitative data on help‐seeking or not focusing on student populations.

**Table 1:** Eligibility criteria

PICOS element	Inclusion criteria	Exclusion criteria
Population	College students	Noncollege student populations
	Students with mental health conditions (meeting DSM/ICD criteria for a disorder—current, 12‐month, or lifetime) or showing high distress on validated tools (e.g., PHQ‐9, GAD‐7)	College students without mental health conditions
Intervention	Help‐seeking behaviors from formal sources (e.g., qualified mental health professionals, general practitioners)	Help‐seeking from informal sources (e.g., family, friends)
	Help‐seeking intentions (any future actions related to mental health service use, including likelihood of using and perceived need)	
	Barriers to help‐seeking (if reasons for not seeking help were provided)	
Comparator outcomes	No specific comparator is required	
	Prevalence of help‐seeking behaviors	Purely qualitative outcomes
	Prevalence of help‐seeking intentions	Insufficient data for prevalence calculation
	Prevalence of barriers to help‐seeking	
Study design	Empirical studies	Experimental studies
	Peer‐reviewed publications	Qualitative studies
		Nonpeer reviewed sources

**Figure 1:**
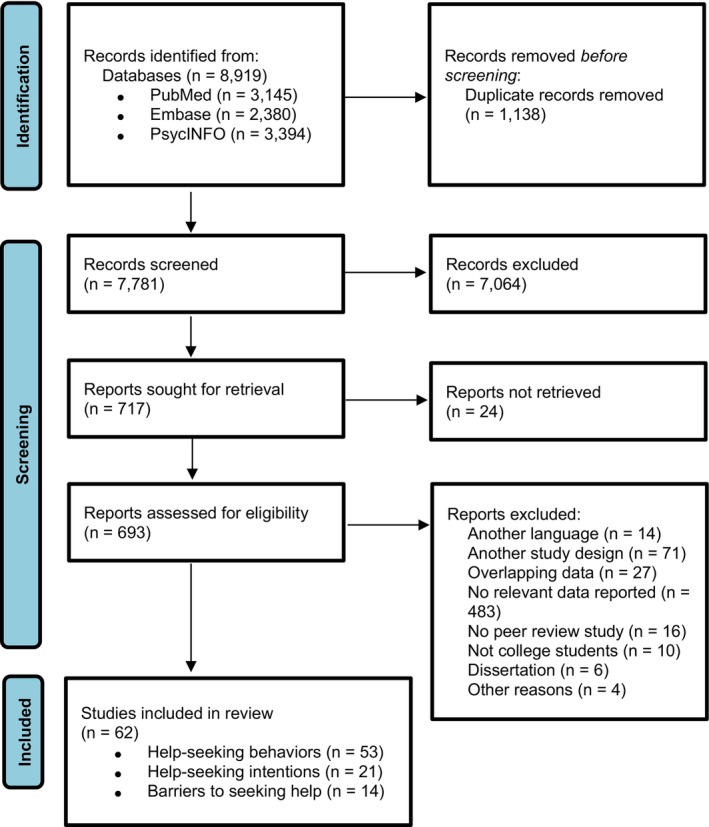
PRISMA flowchart

Upon retrieving all relevant studies and removing duplicates, two researchers (different combinations of YA and JP, RZ, CG, LAF, FCR, SNU, and OR) independently screened the titles and abstracts based on the criteria, followed by a full‐text review. Discrepancies among the researchers were resolved through discussion with the senior researcher (PC). The systematic review process can be seen in Figure 1.

### Data extraction

Two researchers (different combinations of YA and JP, RZ, CG, LAF, FCR, and OR) independently extracted relevant information from the full texts using a standardized data extraction sheet. The coded information included:

1. Characteristics of studies: publication year, country of study, and design.
2. Characteristics of participants: mean age, academic level (undergraduate, graduate, mixed, or other), percentage of female participants, recruitment strategy, and whether compensation was provided for participation.
3. Information on mental health problems: definition of mental health conditions, validation tools used, and cutoff score.
4. Characteristics of outcome: details regarding help‐seeking behaviors or intentions (measurement, scoring system, and time frame), details of barriers reported by students (the question of help‐seeking barriers, specific reason for not seeking help, measurement).
5. Outcomes: For intentions and behaviors, the number of students indicating help‐seeking intentions/behaviors and the total number of students that endorsed mental health conditions were extracted. For barriers, the number of students who reported each reason and the total number of students were extracted. If studies presented data in percentages (e.g., the percentage of students with mental health conditions or those actively seeking help), these percentages were converted into raw numbers. If crucial data, either as absolute counts or percentages, were missing from a study, the respective authors were contacted for clarification.

### Quality assessment

The quality assessment of each study was independently conducted by two researchers (different combinations of YA and JP, RZ, CG, LAF, FCR, SNU, and OR) using a modified version of the Newcastle‐Ottawa Scale (Mortier et al., 2018; Wells et al., 2021). This adapted version includes five criteria: sample representativeness, sample size, comparability between respondents and nonrespondents, clarity in ascertaining help‐seeking intentions/behaviors or reasons for avoidance among college students with mental health conditions, and the quality of descriptive statistics reporting (full scoring details are provided in Appendix S2). Each criterion is scored as 0 if it does not meet the quality criteria and 1 if it meets the criteria, resulting in an overall score ranging from 0 to 5. Studies that achieved a score of 3 and above were categorized as low risk of bias. Any disagreements between the raters were resolved through discussion.

### Data synthesis and statistical analysis

A series of meta‐analyses were conducted for help‐seeking behaviors, help‐seeking intentions, and each individual barrier to help‐seeking. Initially, studies examining help‐seeking behaviors in various mental health conditions were pooled. For studies focusing on specific conditions, such as depression, data were aggregated for that mental health condition. Additionally, studies were classified based on reported help‐seeking behaviors for specific mental health conditions, allowing analysis by time frame (i.e., current, 3‐month, 12‐month, or lifetime rates) and type of help sought (i.e., medication or psychotherapy/counseling).

Separate meta‐analyses were conducted for help‐seeking intentions, categorized by different mental health conditions, including depression, anxiety, alcohol and drug use problems, suicidal thoughts and behaviors, and psychological distress. Additionally, two separate meta‐analyses were conducted based on the conceptualizations of help‐seeking intentions: likelihood of seeking help and perceived need.

When pooling studies investigating help‐seeking barriers, it became evident that these barriers were reported heterogeneously across studies. To address this, common themes of help‐seeking barriers, as identified in the literature, were systematically classified (Gulliver et al., 2010). These categories include (1) preference for self‐management of the issues, (2) time constraints, (3) perceived lack of necessity, (4) financial concerns, (5) limited awareness of where to seek assistance, (6) privacy apprehensions, (7) preference for seeking informal support (e.g., from family or friends), (8) social stigma, (9) fear of receiving undesired treatment, (10) challenges in accessing services, (11) worries about implications on academic records, (12) perceptions of treatment ineffectiveness, (13) previous negative treatment experiences, and (14) cultural insensitivity exhibited by service providers. Separate analyses were then conducted for each category, combining data from studies that reported on these specific areas.

All analyses were performed using RStudio version 4.3.0 with the packages “dmetar,” “metafor,” and “meta” (Balduzzi, Rücker, & Schwarzer, 2019; Harrer, Cuijpers, et al., 2019; Viechtbauer, 2010). The “metaprop” function of the “meta” package was used for pooling proportions. Before the meta‐analysis was performed, the raw proportions were logit transformed. Due to the expected wide variety across studies, a random effects model, based on the DerSimonian‐Laird estimator, was used for pooling (DerSimonian & Laird, 1986). *I* ^2^ was calculated to explore between‐study heterogeneity. Heterogeneity was interpreted as low, moderate, or high based on *I* ^2^ values of 25%, 50%, and 75%, respectively (Higgins & Thompson, 2002). The 95% prediction intervals were calculated to indicate the expected range of true effects that may occur in future research. Subgroup analyses were performed to examine the potential reasons for heterogeneity in the random effect models. Subgroups were defined based on differences in country income level (high‐income vs. low‐middle‐income), risk of bias assessment (low risk vs. high risk), student type (undergraduate students vs. mixed students vs. not specified or other), and compensation (yes vs. no or not specified). Publication bias was investigated using funnel plots and Peters' regression test (Peters, 2006). Furthermore, sensitivity analyses were performed by excluding outliers.

## Results

### Study selection

Initially, 8,919 studies were identified. After removing 1,138 duplicates, the titles and abstracts of 7,781 studies were screened, resulting in the exclusion of 7,088 studies. The remaining 693 studies underwent further screening, resulting in the final inclusion of 53 studies on help‐seeking behaviors, 21 on help‐seeking intentions, and 14 on barriers to seeking help. In total, 631 studies were excluded for the following reasons: 10 targeted noncollege student populations, 71 used different study designs, 16 were not peer‐reviewed articles, 6 were dissertations, 483 did not report relevant data, 14 were written in languages other than those included in the review, 27 had overlapping data, and 4 were excluded for other specific reasons. The flow chart of the selection process can be found in Figure 1. References of included studies are presented in Table S1.

### Study characteristics

Of the 53 studies on help‐seeking behaviors (Table S2), most (87%) were carried out in high‐income countries, whereas seven studies were conducted in low‐ and middle‐income countries. Forty‐five were cross‐sectional studies, and eight were longitudinal studies. A total of 22 studies included undergraduate students, 26 studies included both undergraduate and graduate students, and 5 studies included other student samples or unspecified. Females typically made up more than half of the sample in most studies. Twenty‐one studies offered compensation to participants during data collection. The most frequently reported mental health problems were depression and anxiety (42%). Of the 21 studies on help‐seeking intentions (Table S3), two were conducted outside high‐income countries (Mexico and Ethiopia). Thirteen studies investigated the perceived need for help, five evaluated the likelihood of seeking help, and three explored interests in treatment.

### Results of the meta‐analysis

#### Help‐seeking behaviors

**Figure 2:**
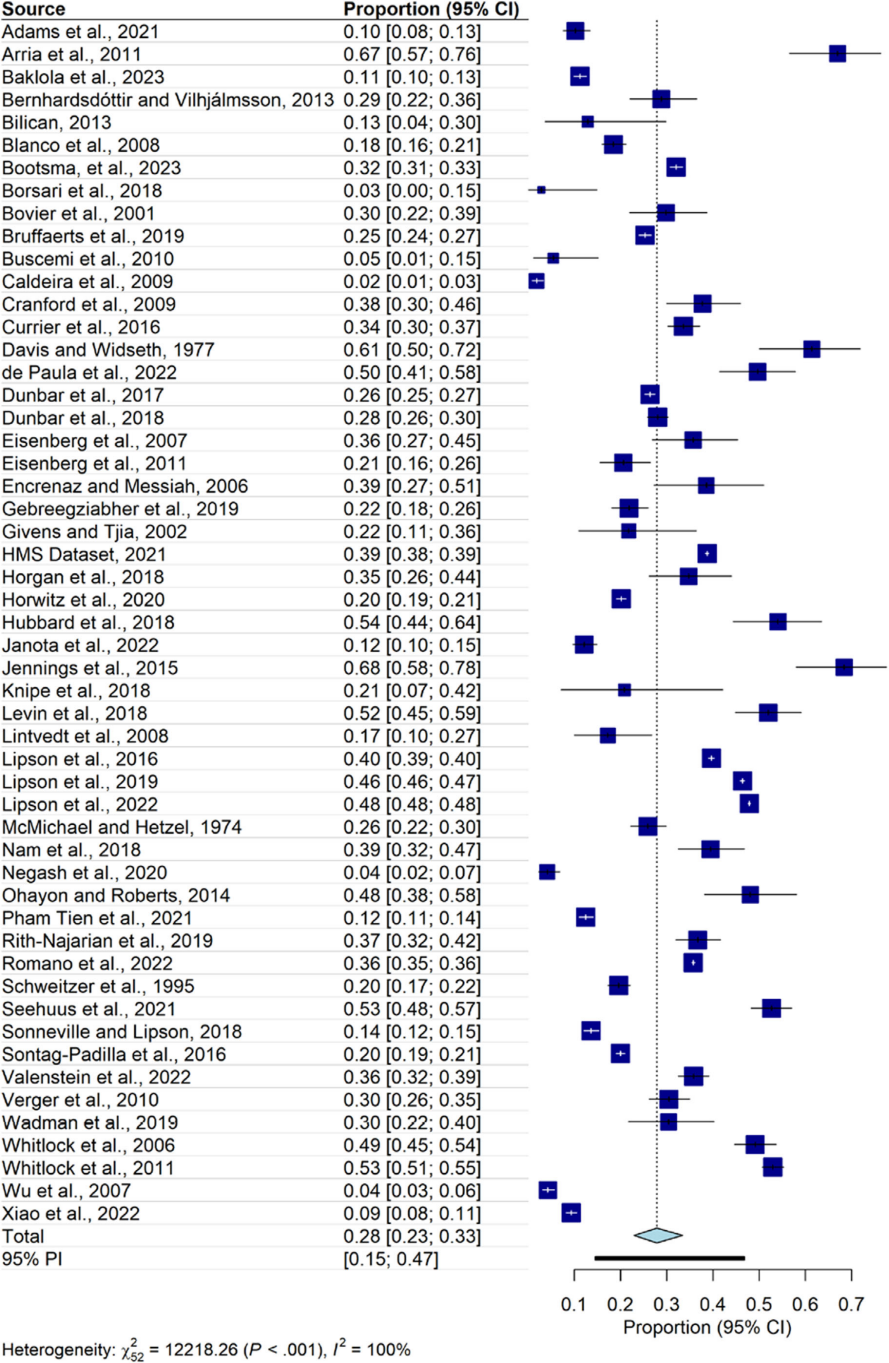
Forest plot of help‐seeking behaviors

Fifty‐three studies were included in the analysis of help‐seeking behaviors (435,768 students, with 179,915 identified with mental health conditions). The results showed that 28% ([95% CI: 23%–33%]; prediction interval: 15–17) of the students with mental health conditions received current to lifetime treatment. Heterogeneity was very high (*I* ^2^ = 99.6% [95% CI: 99.5%–99.6%]) (Table 2, Figure 2). After removing 30 outliers, the pooled prevalence rate remained comparable (30% [95% CI: 27%–32%]; prediction interval: 22–39), but heterogeneity was still very high at 86.5% [95% CI: 81%–90.4%]. Peters' regression analysis revealed no indication of significant asymmetry of the funnel plot (*t* = −0.99, *p* = 0.33) (Figure S1).

**Table 2:** Prevalence rates of help‐seeking behaviors among college students

	No. of studies	No. HSB	Total no.	Prevalence (%)	95% CI	*I* ^2^ [95% CI]	*p* ^b^	Prediction interval
All studies	53	179,915	435,768	28	23–33	99.6% [99.5%–99.6%]		15–47
After removing outliers	23	6,168	21,643	30	27–32	86.5% [81.0%–90.4%]		22–39
Mental health condition								
Depression	24	41,666	101,258	34	27–43	98.7% [98.5%–98.9%]		20–52
Anxiety	12	18,872	47,811	34	22–48	98.9% [98.7%–99.1%]		9–72
Suicidal thoughts and behaviors	10	14,467	37,730	33	22–47	99.4% [99.3%–99.5%]		8–74
Psychological distress	12	4,078	18,248	24	16–35	97.5% [96.6%–98.1%]		10–47
Alcohol and/or drug use problems	8	347	3,390	12	4–28	97.2% [95.9%–98.0%]		0.7–70
Eating disorders	4	24,796	70,509	27	10–55	99.1% [98.7%–99.4%]		0.7–95
Subgroups analyses								
Country income level								
High income	46			30	25–36	99.6% [99.5%–99.6%]	**.01**	
Low‐middle income	7			14	6–29	97.3% [95.9%–98.2%]		
Risk of bias assessment^a^								
Low risk	30			27	21–33	99.7% [99.7%–99.8%]	.97	
High risk	22			27	17–39	96.3% [95.25%–97.05%]		
Type of student								
Undergraduate students	22			23	14–34	98.2% [97.9%–98.5%]	.25	
Mixed (undergraduate and graduate students)	26			32	27–37	99.7% [99.7%–99.7%]		
Compensation								
Yes	21			35	26–45	99.6% [99.6%–99.7%]	**.03**	
No or N.S.	32			24	18–30	99.2% [99.1%–99.3%]		

HSB, help‐seeking behaviors; N.S., not specified. Bolded values represent statistically significant differences (*p*  <  .05).

^a^ Data sets obtained from personal communication with the authors were not excluded from this subgroup analysis.

^b^ *p*‐values indicate whether the difference between subgroups is statistically significant.

Separate analyses were also performed for each specific mental health condition (Table 2). These analyses showed that 34% of students with depression [95% CI: 27%–43%], 34% of students with anxiety [95% CI: 22%–48%], and 33% of students with suicidal thoughts and behaviors [95% CI: 22%–47%] sought help. Students with alcohol and/or drug use problems demonstrated a notably low tendency to seek help (i.e., only 12% [95% CI: 4%–28%]). Furthermore, separate analyses were conducted for different time points of help‐seeking behaviors and different types of help (i.e., medication and psychotherapy/counseling); the results are reported in Table S4.

The subgroup analysis of help‐seeking behaviors did not show significant associations between prevalence rates and student type (i.e., undergraduate vs. mixed; *p* = .25) and risk of bias (i.e., low vs. high; *p* = .97). However, the prevalence rate was significantly associated with country income level (higher in high‐income countries compared to low‐ and middle‐income countries; *p* = .01) and compensation (higher in studies offering compensation compared to those without compensation or unspecified; *p* = .03) (Table 2).

#### Help‐seeking intentions

**Figure 3:**
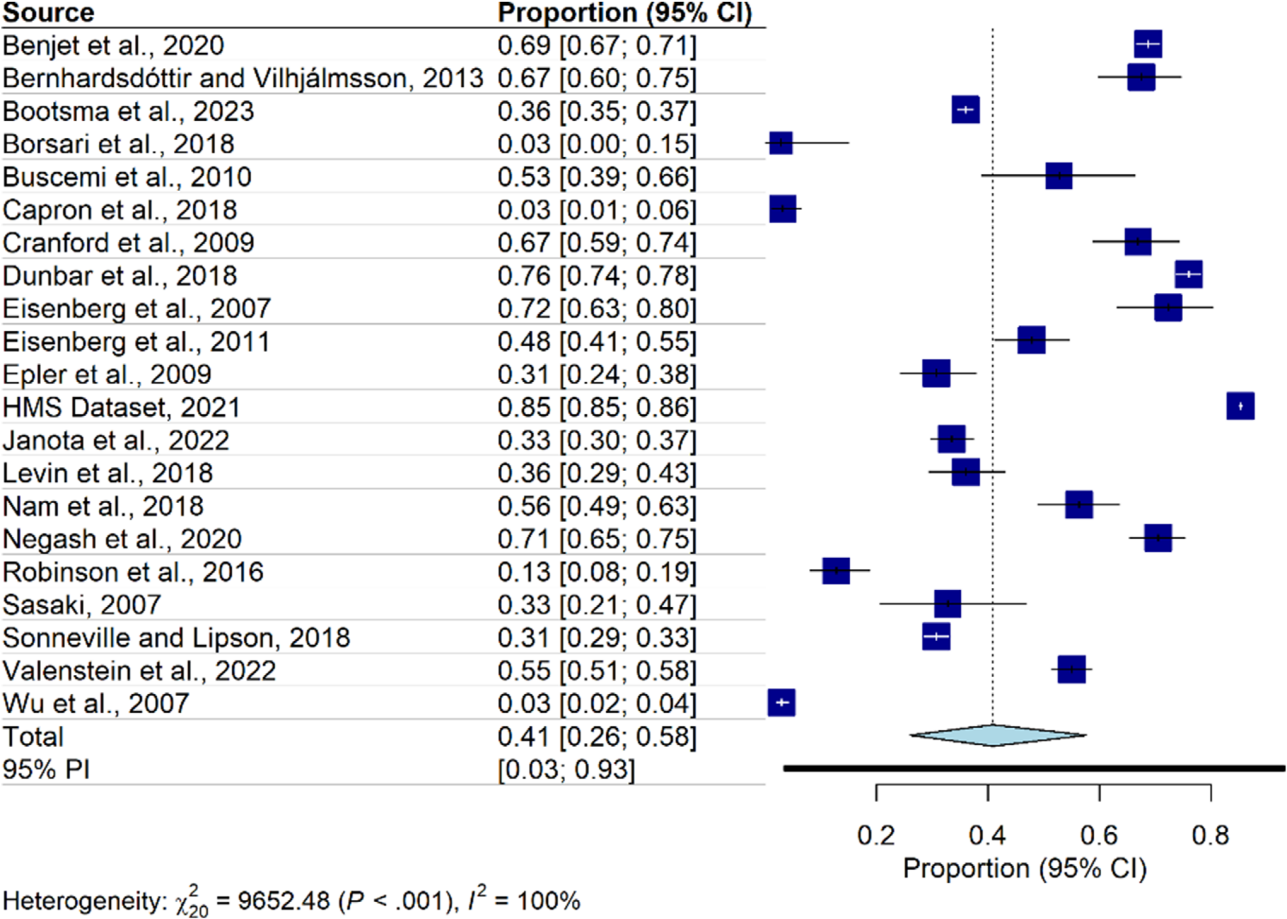
Forest plot of help‐seeking intentions

The primary analysis of help‐seeking intentions included data from 21 studies. Overall, help‐seeking intentions were present in 41% of college students ([95% CI: 26%–58%]; prediction interval: 4 to 93), with very high heterogeneity (*I* ^2^ = 99.8%; [95% CI: 99.7%–99.8%]). After removing the 10 outliers, the prevalence of help‐seeking intentions did not change (41%; [95% CI: 33%–48%]; prediction interval: 22–63), and heterogeneity remained very high (*I* ^2^ = 95.3%; [95% CI: 93%–96.8%]) (Table 3, Figure 3). Peters' regression analysis did not show any evidence of asymmetry in the funnel plot (*t* = −1.67, *p* = .11) (Figure S2).

**Table 3:** Prevalence rates of help‐seeking intentions among college students

	No. of studies	No. HSI	Total no.	Prevalence (%)	95% CI	*I* ^2^ [95% CI]	Prediction interval
All studies	21	62,456	80,161	41	26–58	99.8% [99.7%–99.8%]	4–93
After removing outliers	10	2,802	7,108	41	33–48	95.3% [93.0%–96.8%]	22–63
Mental health condition							
Depression	6	42,930	49,110	67	44–84	97.6% [96.4%–98.4%]	8–98
Anxiety	5	37,920	42,562	69	39–89	98.2% [97.2%–98.8%]	3–99
Alcohol or drug use problems	5	170	1,660	14	2–54	97.9% [96.7%–98.6%]	0.1–96
Suicidal thoughts and behaviors	4	25,402	29,005	68	23–94	99.7% [99.7%–99.8%]	0.04–99.99
Psychological distress	4	1,515	2,261	48	9–90	98.8% [98.2%–99.2%]	0.04–99.5

HSI, help‐seeking intentions.

Separate analyses for each specific mental health condition indicated that around 70% of students with depression, anxiety, or suicidal thoughts and behaviors reported help‐seeking intentions. Only 14% of students with alcohol and/or drug use problems reported help‐seeking intentions (Table 3). Furthermore, the results of separate analyses for prevalence rates of help‐seeking intentions, categorized by type of help (perceived need only and likelihood of seeking help), are reported in Table S5.

#### Barriers to mental health help‐seeking

Fourteen studies identified barriers to help‐seeking. The analysis revealed that the barriers most frequently cited were: preference for self‐management of the problem at 41% ([95% CI: 26%–59%]; prediction interval: 17–71), lack of time at 41% ([95% CI: 27%–56%]; prediction interval: 4–91), lack of knowledge about where to seek help at 35% ([95% CI: 20%–53%]; prediction interval: 18–57), and lack of perceived need at 34% ([95% CI: 19%–54%]; prediction interval: 3–90). All additional reasons students have cited for not seeking help are detailed in Table 4.

**Table 4:** Prevalence rates of barriers to mental health help‐seeking among college students

	No. of studies	No. SWB	Total no.	Prevalence (%)	95% CI	*I* ^2^ [95% CI]	Prediction interval
Preference for self‐management of the problem	7	8,934	18,085	41	26–59	98.9% [98.5%–99.2%]	17–71
Lack of time	9	3,120	6,473	41	27–56	99.2% [99.0%–99.4%]	4–91
Lack of knowledge of where to seek help	7	1936	5,823	35	20–53	92.4% [86.8%–95.6%]	18–57
Lack of perceived need	9	3,318	7,900	34	19–54	99.4% [99.3%–99.5%]	3–90
Perceived cost	9	3,014	9,368	28	15–45	99.1% [98.8%–99.3%]	4–78
Privacy concerns	4	1,154	3,896	28	14–47	93.4% [86.3%–96.8%]	3–85
Stigma	11	3,228	9,971	28	17–42	98.5% [98.0%–98.8%]	7–66
Preference of informal help‐seeking (e.g., family or friends)	6	2,638	9,212	26	16–39	99.0% [98.7%–99.3%]	5–72
Difficult to access	9	2096	9,338	23	17–30	94.7% [91.9%–96.5%]	11–43
Fear of unwanted treatment	5	1,294	5,627	22	10–42	92.7% [85.8%–96.2%]	8–47
Concerns about academic record	7	1795	7,633	20	11–34	94.3% [90.5%–96.5%]	9–41
Perceived ineffectiveness	10	2,839	9,947	20	12–33	98.3% [97.8%–98.7%]	5–55
Cultural insensitivity	5	1,049	16,351	8	4–14	98.9% [98.4%–99.2%]	0.61–53
Negative past experience with treatment	4	689	5,714	7	2–21	98.5% [97.5%–99.0%]	0.05–94

No. SWB: number of students with this barrier.

### Quality assessment

Of the 62 studies considered for the three meta‐analyses, 25 were classified as having a high risk of bias (40%) (Table S6). The risk of bias for one study could not be evaluated because the dataset was obtained through personal contact with the authors, making it impossible to evaluate its quality. Among the studies analyzed, 30 did not meet the sample representativeness criteria (48%), 27 did not meet the sample size requirements (44%), 54 reported low response rates (87%), and 19 did not report descriptive statistics (31%). However, all studies used validated tools to identify symptoms of mental disorders.

## Discussion

This systematic review and meta‐analysis examined the prevalence of help‐seeking behaviors, intentions, and self‐reported reasons for not seeking help among college students with a mental disorder and/or significant distress. In total, 62 studies were included. Among the 53 included studies reporting help‐seeking behaviors, approximately one‐third of college students actively sought help from mental health professionals. However, among the 21 studies reporting help‐seeking intentions, nearly half of the students intended to seek help. Among the 14 studies discussing barriers to help‐seeking, nearly half preferred to solve their mental health problems by themselves. Other commonly reported barriers were a lack of time, knowledge of where to seek help, perceived lack of need, and concerns about high costs, privacy, and stigma.

The finding that only 28% of college students with mental health problems actively seek professional help is in line with recent meta‐analysis evidence, which reported a pooled proportion of 35% for the use of mental health services by university students (Osborn et al., 2022). The series of separate meta‐analyses resulted in similar proportions of help‐seeking for each mental disorder or mental health condition, such as depression, anxiety, suicidal thoughts and behaviors, psychological distress, or eating disorders; about two‐thirds of college students with those mental disorders did not seek help. Furthermore, the estimated proportion of help‐seeking behavior was even lower in students with alcohol and/or drug use problems, only 12% of whom sought help. This could be because individuals with alcohol use disorders may not recognize their condition as a problem and often do not perceive a need for treatment until their disorders become significantly debilitating (Kaskutas, Weisner, & Caetano, 1997). It is also possibly related to the drinking culture on campuses, where social norms and peer influences may facilitate alcohol consumption and attitudes toward help‐seeking for alcohol‐related problems.

It is also important to note that, if left untreated, mental disorders may evolve into more severe or complex conditions, possibly leading to comorbidity and chronic health conditions (Kessler & Price, 1993; Kisely et al., 2006). This progression makes them more difficult to treat. In the long run, it may cause a heavy burden on society, as individuals with mental disorders may experience decreased productivity and require additional financial support (GBD 2019 Mental Disorders Collaborators, 2022). Therefore, it is important to make efforts to encourage college students with mental disorders to seek appropriate and timely services and treatments. The meta‐analysis on help‐seeking intentions indicated that almost half of college students with mental disorders have intentions to seek help. The highest prevalence of help‐seeking intentions (70%) was found in students with depression, anxiety, or suicidal thoughts and behaviors. Conversely, the lowest prevalence of help‐seeking intentions (14%) was found in students facing alcohol and/or drug use issues. However, although college students expressed high intentions overall, they did not translate these intentions into actions to seek professional help, as evidenced by their low rates of help‐seeking behavior (12%). This result is in line with a previous study, which found that help‐seeking intentions had no direct effect on help‐seeking behavior (Doll et al., 2021).

The perceived barriers identified in the results could inform targeted strategies to increase help‐seeking behavior for those in need. Approximately half of college students prefer to handle mental disorders themselves without outside help, which is not surprising. Self‐reliance in addressing mental health issues has been recognized as a barrier, as highlighted in prior systematic reviews focused on young individuals (Gulliver et al., 2010; Lui et al., 2022). This might be because of the desire for autonomy and independence during the developmental transition to adulthood. Studies conducted in high‐income countries found that young people hold the belief that they should be capable of addressing challenges on their own, even when those problems involve psychological distress (Carlton & Deane, 2000; Deane, Wilson, & Ciarrochi, 2001). Considering this finding, it is plausible that self‐help interventions, particularly internet‐based interventions, could be well suited for students who prefer to deal with mental health problems independently. These interventions involve psychological interventions based primarily on self‐help material delivered over the Internet (Karyotaki et al., 2018).

Lack of time was equally as prevalent as self‐reliance preferences among college students. Furthermore, approximately one‐third reported that professional help such as a qualified mental health professional would be cost‐prohibitive. Considering the lack of accessibility (e.g., time, cost), providing scalable digital mental health interventions, such as online therapy and mental health apps, may potentially enhance treatment uptake for students with these concerns, since they offer a convenient and accessible platform for addressing mental health needs and are effective for college students with mental disorders (Harrer, Adam, et al., 2019; Harrer, Cuijpers, et al., 2019).

Surprisingly, even when mental health services are available (Eisenberg et al., 2012), a lack of knowledge about potential sources of help is still a barrier for 35% of students. Furthermore, more than one‐third of the students reported low perceived need, suggesting that they did not view their mental disorders as serious enough to take action and therefore did not feel the need for professional help. Those two common barriers might indicate poor mental health literacy among college students, including recognizing mental health problems, understanding risks, causes, and treatments, and knowing how to seek information and services on mental health (Jorm et al., 1997). Colleges can address these challenges by organizing informative campaigns, including psychoeducation workshops, peer support programs, and digital platforms, to increase students' awareness about available mental health resources. However, further research is needed to explore effective strategies for increasing mental health literacy among college students.

Although many efforts over the past years to close the gaps in mental health care have been made, the proportion of students seeking treatment remains low. This problem highlights the need for new intervention modes and for effective implementation strategies. Insights can be drawn from theoretical frameworks for help‐seeking. According to Andersen's behavioral model, factors at the individual, social, and structural levels shape help‐seeking behaviors (Andersen, 1995). A recent meta‐analysis found that certain sociodemographic characteristics, like sex and gender, are associated with the treatment‐seeking among college students (Pei et al., 2024). Future studies need to identify and overcome barriers throughout the help‐seeking framework, investigating multifaced nature of help‐seeking behaviors and testing existing models such as Anderson's in the university context.

Several limitations to the current review need to be considered. First, the focus is mainly on the general population of college students' help‐seeking for mental disorders. However, specific student populations, such as international students and those from diverse ethnic backgrounds, may have even higher rates of mental disorders, lower rates of help‐seeking behaviors and intentions, and greater barriers to seeking help (Crockett et al., 2024; Crockett, Martínez, & Caviedes, 2022; Eylem et al., 2020; Jamilah et al., 2021; Pei et al., 2024).

Second, only self‐reported barriers were explored, which may not cover all potentially influential factors affecting help‐seeking. It is important to go beyond individual barriers and consider contextual factors, particularly university characteristics. For example, a study revealed a negative relationship between perceived stigma and smaller institutions that have greater admission selectivity (Gaddis, Ramirez, & Hernandez, 2020). In smaller or highly competitive institutions, students might be reluctant to use mental health services due to reduced anonymity, or they fear that seeking mental health support could have a potential negative impact on their academic records. Future studies could explore variations in help‐seeking barriers across different universities.

Third, diverse approaches were used to report barriers across included studies, which introduced potential omissions of reasons for not seeking help when classifying them into common domains. Fourth, extremely high heterogeneity was observed in most analyses, and it remained high even after excluding outliers. This high degree of heterogeneity is consistent with findings from previous meta‐analyses of proportions (Winsper et al., 2020; Zhao, Amarnath, Karyotaki, Struijs, & Cuijpers, 2022).

Another limitation is that most included studies (87%) were conducted in high‐income countries. Less is known about situations in low‐ and middle‐income countries (LMICs). Resources for utilizing formal mental health services could be different between these regions (Ito, Setoya, & Suzuki, 2012). Additionally, increasing help‐seeking behaviors also poses challenges for care services. In many colleges, the size of counseling centers and counseling staff cannot meet the rising demand (Abrams, 2022). Future research on attitudes toward the services is needed. Finally, the cross‐sectional nature of the included studies introduces another limitation, as the sampling frameworks and assessment methods varied widely among the studies. Therefore, the proportion of help‐seeking behaviors, intentions, and reasons for not seeking help should be interpreted with caution.

## Conclusion

This systematic review and meta‐analysis reveal an increasing trend in intentions to seek help among college students with mental health problems. However, the prevalence of formal help‐seeking behaviors remains low. Additionally, prominent barriers include self‐reliance, lack of time, limited awareness of available resources, and lack of perceived need. The findings suggest that treatment engagement can be increased through psychoeducation programs, accessible support resources, and evidence‐based self‐help materials and digital interventions. Further research is needed to explore other influential factors that affect help‐seeking behaviors and barriers to seeking help, including individual sociodemographic factors and contextual factors, and further investigate potential interventions to promote help‐seeking.

## Ethical considerations

As this study is a meta‐analysis, it does not require ethical approval.

**Key points**

- This is the first meta‐analysis to explore the rates of help‐seeking intentions, behaviors, and self‐reported barriers among college students with mental health problems.
- This meta‐analysis of 62 studies found that 41% of college students with mental health problems consider using formal help, whereas 28% actively seek formal help. The main reasons for not seeking help were self‐reliance (41%), lack of time (41%), limited awareness of available resources (35%), and lack of perceived need (34%).
- Practitioners should acknowledge the disparity between students' mental health needs and their help‐seeking behaviors.
- Efforts should be directed toward addressing both personal and systemic barriers to accessing mental health services on college campuses.
- Further research is needed to develop tailored interventions that promote help‐seeking behaviors among college students.

## Supporting information


**Appendix S1.** Full search strings.
**Appendix S2.** The modified Newcastle‐Ottawa Scale.
**Figure S1.** Funnel plot for prevalence rates of help‐seeking behaviors among college students.
**Figure S2.** Funnel plot for prevalence rates of help‐seeking intentions among college students.
**Table S1.** References of included studies and their alignment with help‐seeking behaviors, help‐seeking intentions, and barriers.
**Table S2.** Key characteristics for studies reporting help‐seeking behaviors.
**Table S3.** Key characteristics for studies reporting help‐seeking intentions.
**Table S4.** Prevalence rates of help‐seeking behaviors by time point and type of help.
**Table S5.** Prevalence rates of help‐seeking intentions by type of help.
**Table S6.** Risk of bias assessment of included studies.

## Data Availability

Data are presented in the manuscript and Supporting Information. The raw data and R codes used for the meta‐analyses are available upon request from the corresponding author.

## References

[jcpp14145-bib-0001] Abassi, B. , Fekih‐Romdhane, F. , Maktouf, H. , & Moalla, I. (2021). The relationship between stigma and help‐seeking intentions in college students. European Psychiatry, 64, S393.

[jcpp14145-bib-0002] Abrams, Z. (2022). Student mental health is in crisis: Campuses are rethinking their approach. Monitor on Psychology, 53, 60.

[jcpp14145-bib-0003] Altamura, A.C. , Dell'Osso, B. , Berlin, H.A. , Buoli, M. , Bassetti, R. , & Mundo, E. (2010). Duration of untreated illness and suicide in bipolar disorder: A naturalistic study. European Archives of Psychiatry and Clinical Neuroscience, 260, 385–391. 19911248 10.1007/s00406-009-0085-2

[jcpp14145-bib-0004] Altamura, A.C. , Santini, A. , Salvadori, D. , & Mundo, E. (2005). Duration of untreated illness in panic disorder: A poor outcome risk factor? Neuropsychiatric Disease and Treatment, 1, 345–347. 18568114 PMC2424121

[jcpp14145-bib-0005] Andersen, R.M. (1995). Revisiting the behavioral model and access to medical care: Does it matter? Journal of Health and Social Behavior, 36, 1–10. 7738325

[jcpp14145-bib-0006] Apolinário‐Hagen, J. , Hennemann, S. , Kück, C. , Wodner, A. , Geibel, D. , Riebschläger, M. , & Breil, B. (2020). Exploring user‐related drivers of the early acceptance of certified digital stress prevention programs in Germany. Health Services Insights, 13, 1–11. 10.1177/1178632920911061 PMC7074489 32206013

[jcpp14145-bib-0007] Arnett, J.J. (2000). Emerging adulthood: A theory of development from the late teens through the twenties. American Psychologist, 55, 469–480. 10842426

[jcpp14145-bib-0008] Auerbach, R.P. , Mortier, P. , Bruffaerts, R. , Alonso, J. , Benjet, C. , Cuijpers, P. , … & WHO WMH‐ICS Collaborators . (2018). The WHO World Mental Health Surveys International College Student Project: Prevalence and distribution of mental disorders. Journal of Abnormal Psychology, 127, 623–638. 30211576 10.1037/abn0000362 PMC6193834

[jcpp14145-bib-0009] Balduzzi, S. , Rücker, G. , & Schwarzer, G. (2019). How to perform a meta‐analysis with R: A practical tutorial. Evidence‐Based Mental Health, 22, 153–160. 31563865 10.1136/ebmental-2019-300117 PMC10231495

[jcpp14145-bib-0010] Bruffaerts, R. , Mortier, P. , Kiekens, G. , Auerbach, R.P. , Cuijpers, P. , Demyttenaere, K. , & Kessler, R.C. (2018). Mental health problems in college freshmen: Prevalence and academic functioning. Journal of Affective Disorders, 225, 97–103. 28802728 10.1016/j.jad.2017.07.044 PMC5846318

[jcpp14145-bib-0011] Buchanan, J.L. (2012). Prevention of depression in the college student population: A review of the literature. Archives of Psychiatric Nursing, 26, 21–42. 22284078 10.1016/j.apnu.2011.03.003

[jcpp14145-bib-0012] Carlton, P.A. , & Deane, F.P. (2000). Impact of attitudes and suicidal ideation on adolescents' intentions to seek professional psychological help. Journal of Adolescence, 23, 35–45. 10700370 10.1006/jado.1999.0299

[jcpp14145-bib-0013] Cramer, K.M. (1999). Psychological antecedents to help‐seeking behavior: A reanalysis using path modeling structures. Journal of Counseling Psychology, 46, 381–387.

[jcpp14145-bib-0014] Crockett, M.A. , Martínez, V. , & Caviedes, P. (2022). Barriers and facilitators to mental health help‐seeking and experiences with service use among LGBT+ university students in Chile. International Journal of Environmental Research and Public Health, 19, 16520. 36554401 10.3390/ijerph192416520 PMC9779696

[jcpp14145-bib-0015] Crockett, M.A. , Martínez, V. , Mac‐Ginty, S. , Langer, Á.I. , Gaete, J. , Núñez, D. , & Léniz, I. (2024). Mental health service use and barriers to help‐seeking among LGBTQ+ first‐year college students in Chile. International Journal of LGBTQ+ Youth Studies, 1–22. 10.1080/19361653.2024.2347962

[jcpp14145-bib-0016] Cuijpers, P. , Cristea, I.A. , Ebert, D.D. , Koot, H.M. , Auerbach, R.P. , Bruffaerts, R. , & Kessler, R.C. (2016). Psychological treatment of depression in college students: A metaanalysis. Depression and Anxiety, 33, 400–414. 26682536 10.1002/da.22461 PMC4846553

[jcpp14145-bib-0017] Deane, F.P. , Wilson, C.J. , & Ciarrochi, J. (2001). Suicidal ideation and help‐negation: Not just hopelessness or prior help. Journal of Clinical Psychology, 57, 901–914. 11406803 10.1002/jclp.1058

[jcpp14145-bib-0018] DerSimonian, R. , & Laird, N. (1986). Meta‐analysis in clinical trials. Controlled Clinical Trials, 7, 177–188. 3802833 10.1016/0197-2456(86)90046-2

[jcpp14145-bib-0019] Doll, C.M. , Michel, C. , Rosen, M. , Osman, N. , Schimmelmann, B.G. , & Schultze‐Lutter, F. (2021). Predictors of help‐seeking behaviour in people with mental health problems: A 3‐year prospective community study. BMC Psychiatry, 21, 1–11. 34479537 10.1186/s12888-021-03435-4 PMC8414662

[jcpp14145-bib-0020] Ebert, D.D. , Franke, M. , Kählke, F. , Küchler, A.‐M. , Bruffaerts, R. , Mortier, P. , … & Baumeister, H. (2019). Increasing intentions to use mental health services among university students. Results of a pilot randomized controlled trial within the World Health Organization's World Mental Health International College Student Initiative. International Journal of Methods in Psychiatric Research, 28, e1754. 30456814 10.1002/mpr.1754 PMC6877244

[jcpp14145-bib-0021] Ebert, D.D. , Mortier, P. , Kaehlke, F. , Bruffaerts, R. , Baumeister, H. , Auerbach, R.P. , … & Kessler, R.C. (2019). Barriers of mental health treatment utilization among first‐year college students: First cross‐national results from the WHO World Mental Health International College Student Initiative. International Journal of Methods in Psychiatric Research, 28, e1782. 31069905 10.1002/mpr.1782 PMC6522323

[jcpp14145-bib-0022] Eisenberg, D. , Hunt, J. , & Speer, N. (2012). Help seeking for mental health on college campuses: Review of evidence and next steps for research and practice. Harvard Review of Psychiatry, 20, 222–232. 22894731 10.3109/10673229.2012.712839

[jcpp14145-bib-0023] Eisenberg, D. , Hunt, J. , Speer, N. , & Zivin, K. (2011). Mental health service utilization among college students in the United States. The Journal of Nervous and Mental Disease, 199, 301–308. 21543948 10.1097/NMD.0b013e3182175123

[jcpp14145-bib-0024] Eylem, O. , de Wit, L. , van Straten, A. , Steubl, L. , Melissourgaki, Z. , Danışman, G.T. , … & Cuijpers, P. (2020). Stigma for common mental disorders in racial minorities and majorities a systematic review and meta‐analysis. BMC Public Health, 20, 1–20. 32513215 10.1186/s12889-020-08964-3 PMC7278062

[jcpp14145-bib-0025] Gaddis, S.M. , Ramirez, D. , & Hernandez, E.L. (2020). Variations in endorsed and perceived mental health treatment stigma across U.S. higher education institutions. Stigma and Health, 5, 323–330.

[jcpp14145-bib-0026] GBD 2019 Mental Disorders Collaborators . (2022). Global, regional, and national burden of 12 mental disorders in 204 countries and territories, 1990–2019: A systematic analysis for the Global Burden of Disease Study 2019. The Lancet Psychiatry, 9, 137–150. 35026139 10.1016/S2215-0366(21)00395-3 PMC8776563

[jcpp14145-bib-0027] Gulliver, A. , Griffiths, K.M. , & Christensen, H. (2010). Perceived barriers and facilitators to mental health help‐seeking in young people: A systematic review. BMC Psychiatry, 10, 113. 21192795 10.1186/1471-244X-10-113 PMC3022639

[jcpp14145-bib-0028] Harrer, M. , Adam, S.H. , Baumeister, H. , Cuijpers, P. , Karyotaki, E. , Auerbach, R.P. , & Ebert, D.D. (2019). Internet interventions for mental health in university students: A systematic review and meta‐analysis. International Journal of Methods in Psychiatric Research, 28(2), e1759. 10.1002/mpr.1759 30585363 PMC6877279

[jcpp14145-bib-0029] Harrer, M. , Cuijpers, P. , Furukawa, T. , & Ebert, D.D. (2019). dmetar: Companion R package for the guide ‘doing meta‐analysis in R’. R package version 0.0.9000. Available from: https://dmetar.protectlab.org/.

[jcpp14145-bib-0030] Higgins, J.P.T. , & Thompson, S.G. (2002). Quantifying heterogeneity in a meta‐analysis. Statistics in Medicine, 21, 1539–1558. 12111919 10.1002/sim.1186

[jcpp14145-bib-0031] Hunt, J. , & Eisenberg, D. (2010). Mental health problems and help‐seeking behavior among college students. Journal of Adolescent Health, 46, 3–10. 10.1016/j.jadohealth.2009.08.008 20123251

[jcpp14145-bib-0063] Hysenbegasi, A. , Hass, S. L. , & Rowland, C. R. (2005). The impact of depression on the academic productivity of university students. Journal of mental health policy and economics, 8 (3), 145–151. 16278502

[jcpp14145-bib-0032] Ito, H. , Setoya, Y. , & Suzuki, Y. (2012). Lessons learned in developing community mental health care in East and South East Asia. World Psychiatry, 11, 186–190. 23024679 10.1002/j.2051-5545.2012.tb00129.x PMC3449347

[jcpp14145-bib-0033] Jamilah, A. , Haque, M.I. , Muhammad, F. , Harun, M.G.D. , Chowdhury, A.B.M.A. , Akramuzzaman, M. , & Kabir, R. (2021). Depression and associated factors among international students in a private University of Bangladesh. Global Psychiatry Archives, 4, 55–61.

[jcpp14145-bib-0034] Jorm, A.F. , Korten, A.E. , Jacomb, P.A. , Christensen, H. , Rodgers, B. , & Pollitt, P. (1997). “Mental health literacy”: A survey of the public's ability to recognise mental disorders and their beliefs about the effectiveness of treatment. Medical Journal of Australia, 166, 182–186. 9066546 10.5694/j.1326-5377.1997.tb140071.x

[jcpp14145-bib-0035] Karyotaki, E. , Ebert, D.D. , Donkin, L. , Riper, H. , Twisk, J. , Burger, S. , & Andersson, G. (2018). Do guided internet‐based interventions result in clinically relevant changes for patients with depression? An individual participant data meta‐analysis. Clinical Psychology Review, 63, 80–92. 29940401 10.1016/j.cpr.2018.06.007

[jcpp14145-bib-0036] Kaskutas, L.A. , Weisner, C. , & Caetano, R. (1997). Predictors of help seeking among a longitudinal sample of the general population, 1984–1992. Journal of Studies on Alcohol, 58, 155–161. 9065893 10.15288/jsa.1997.58.155

[jcpp14145-bib-0037] Kessler, R.C. , Amminger, G.P. , Aguilar‐Gaxiola, S. , Alonso, J. , & Lee, S. (2007). Age of onset of mental disorders: A review of recent literature. Current Opinion in Psychiatry, 20, 359–364. 17551351 10.1097/YCO.0b013e32816ebc8c PMC1925038

[jcpp14145-bib-0038] Kessler, R.C. , & Price, R.H. (1993). Primary prevention of secondary disorders: A proposal and agenda. American Journal of Community Psychology, 21, 607–633. 8192124 10.1007/BF00942174

[jcpp14145-bib-0039] Kessler, R.C. , Walters, E.E. , & Forthofer, M.S. (1998). The social consequences of psychiatric disorders, III: Probability of marital stability. American Journal of Psychiatry, 155, 1092–1096. 9699699 10.1176/ajp.155.8.1092

[jcpp14145-bib-0040] Kisely, S. , Scott, A. , Denney, J. , & Simon, G. (2006). Duration of untreated symptoms in common mental disorders: Association with outcomes. British Journal of Psychiatry, 189, 79–80. 10.1192/bjp.bp.105.019869 16816310

[jcpp14145-bib-0041] Koelen, J.A. , Mansueto, A.C. , Finnemann, A. , Koning, L. , Heijde, C.M. , Vonk, P. , & Wiers, R.W. (2021). COVID‐19 and mental health among at‐risk university students: A prospective study into risk and protective factors. International Journal of Methods in Psychiatric Research, 31, e1901. 34932250 10.1002/mpr.1901 PMC8886289

[jcpp14145-bib-0042] Lee, J. , Jeong, H.J. , & Kim, S. (2021). Stress, anxiety, and depression among undergraduate students during the COVID‐19 pandemic and their use of mental health services. International Journal of Environmental Research and Public Health, 18, 9891. 33907351 10.1007/s10755-021-09552-y PMC8062254

[jcpp14145-bib-0043] Lisiecka, A. , Chimicz, D. , & Lewicka‐Zelent, A. (2023). Mental health support in higher education during the COVID‐19 pandemic: A case study and recommendations for practice. International Journal of Environmental Research and Public Health, 20, 4969. 36981877 10.3390/ijerph20064969 PMC10049581

[jcpp14145-bib-0044] Lui, J.C. , Sagar‐Ouriaghli, I. , & Brown, J.S.L. (2022). Barriers and facilitators to help‐seeking for common mental disorders among university students: A systematic review. Journal of American College Health, 72(8), 2605–2613. 10.1080/07448481.2022.2119859 36084266

[jcpp14145-bib-0045] Mortier, P. , Cuijpers, P. , Kiekens, G. , Auerbach, R.P. , Demyttenaere, K. , Green, J.G. , & Bruffaerts, R. (2018). The prevalence of suicidal thoughts and behaviours among college students: A meta‐analysis. Psychological Medicine, 48, 554–565. 28805169 10.1017/S0033291717002215

[jcpp14145-bib-0046] Osborn, T.G. , Li, S. , Saunders, R. , & Fonagy, P. (2022). University students' use of mental health services: A systematic review and meta‐analysis. International Journal of Mental Health Systems, 16, 57. 36527036 10.1186/s13033-022-00569-0 PMC9758037

[jcpp14145-bib-0047] Page, M.J. , McKenzie, J.E. , Bossuyt, P.M. , Boutron, I. , Hoffmann, T.C. , Mulrow, C.D. , … & McGuinness, L.A. (2021). The PRISMA 2020 statement: An updated guideline for reporting systematic reviews. BMJ, 372, n71. 33782057 10.1136/bmj.n71 PMC8005924

[jcpp14145-bib-0048] Pandya, A. , & Lodha, P. (2022). Mental health consequences of COVID‐19 pandemic among college students and coping approaches adapted by higher education institutions: A scoping review. SSM – Mental Health, 2, 100122. 35665095 10.1016/j.ssmmh.2022.100122 PMC9148268

[jcpp14145-bib-0049] Pei, J. , Amanvermez, Y. , Vigo, D. , Puyat, J. , Kessler, R.C. , Mortier, P. , & Cuijpers, P. (2024). Sociodemographic correlates of mental health treatment seeking among college students: A systematic review and meta‐analysis. Psychiatric Services, 75, 556–569. 38291886 10.1176/appi.ps.20230414

[jcpp14145-bib-0050] Peters, J.L. (2006). Comparison of two methods to detect publication bias in meta‐analysis. JAMA, 295, 676–680. 16467236 10.1001/jama.295.6.676

[jcpp14145-bib-0051] Priestley, M. , Broglia, E. , Hughes, G. , & Spanner, L. (2022). Student perspectives on improving mental health support Services at University. Counselling and Psychotherapy Research, 22, 197–206.

[jcpp14145-bib-0052] Solmi, M. , Radua, J. , Olivola, M. , Croce, E. , Soardo, L. , Salazar de Pablo, G. , … & Kim, J.Y. (2022). Age at onset of mental disorders worldwide: Large‐scale meta‐analysis of 192 epidemiological studies. Molecular Psychiatry, 27, 281–295. 34079068 10.1038/s41380-021-01161-7 PMC8960395

[jcpp14145-bib-0053] Substance Abuse and Mental Health Services Administration (SAMSA) . (2014). Results from the 2013 National Survey on Drug Use and Health: Mental Health Findings (NSDUH Series H‐49, HHS Publication No. (SMA), pp. 14–4887). Rockville, MD: Substance Abuse and Mental Health Services Administration.

[jcpp14145-bib-0054] Taylor, M.E. , Liu, M. , Abelson, S. , Eisenberg, D. , Lipson, S.K. , & Schueller, S.M. (2024). The reach, effectiveness, adoption, implementation, and maintenance of digital mental health interventions for college students: A systematic review. Current Psychiatry Reports, 26, 683–693. 39392547 10.1007/s11920-024-01545-w PMC11706926

[jcpp14145-bib-0055] Vidourek, R.A. , King, K.A. , Nabors, L.A. , & Merianos, A.L. (2014). Students' benefits and barriers to mental health help‐seeking. Health Psychology and Behavioral Medicine, 2, 1009–1022. 25750831 10.1080/21642850.2014.963586 PMC4346065

[jcpp14145-bib-0056] Viechtbauer, W. (2010). Conducting meta‐analyses in R with the metafor package. Journal of Statistical Software, 36, 1–48.

[jcpp14145-bib-0057] Vogel, D.L. , Wade, N.G. , & Hackler, A.H. (2007). Perceived public stigma and the willingness to seek counseling: The mediating roles of self‐stigma and attitudes toward counseling. Journal of Counseling Psychology, 54, 40–50.

[jcpp14145-bib-0058] Wells, G. , Shea, B. , O'Connell, D. , Peterson, J. , Welch, V. , Losos, M. , & Tugwell, P. (2021). The Newcastle‐Ottawa Scale (NOS) for assessing the quality of nonrandomised studies in meta‐analyses. Available from: https://www.ohri.ca/programs/clinical_epidemiology/oxford.asp

[jcpp14145-bib-0059] White, M.M. , Clough, B.A. , & Casey, L.M. (2018). What do help‐seeking measures assess? Building a conceptualization framework for help‐seeking intentions through a systematic review of measure content. Clinical Psychology Review, 59, 61–77. 29153743 10.1016/j.cpr.2017.11.001

[jcpp14145-bib-0060] Winsper, C. , Bilgin, A. , Thompson, A. , Marwaha, S. , Chanen, A.M. , Singh, S.P. , & Furtado, V. (2020). The prevalence of personality disorders in the community: A global systematic review and meta‐analysis. The British Journal of Psychiatry, 216, 69–78. 31298170 10.1192/bjp.2019.166

[jcpp14145-bib-0061] Yonemoto, N. , & Kawashima, Y. (2023). Help‐seeking behaviors for mental health problems during the COVID‐19 pandemic: A systematic review. Journal of Affective Disorders, 323, 85–100. 36435398 10.1016/j.jad.2022.11.043 PMC9684094

[jcpp14145-bib-0062] Zhao, R. , Amarnath, A. , Karyotaki, E. , Struijs, S.Y. , & Cuijpers, P. (2022). Effects of psychological treatment for depression among people not actively seeking help: A meta‐analysis. Psychological Medicine, 53, 1–12. 10.1017/S0033291722003518 PMC9899569 36404636

