# Supplementary material for: Research Review: Help‐seeking intentions, behaviors, and barriers in college students – a systematic review and meta‐analysis
Source: J Child Psychol Psychiatry. 2025 Mar 12;66(10):1593–605. doi: 10.1111/jcpp.14145 (PMC12447698; doi:10.1111/jcpp.14145)
Supplement: Supplementary file 1 — Appendix S1. Full search strings. Appendix S2. The modified Newcastle‐Ottawa Scale. Figure S1. Funnel plot for prevalence rates of help‐seeking behaviors among college students. Figure S2. Funnel plot for prevalence rates of help‐seeking intentions among college students. Table S1. References of included studies and their alignment with help‐seeking behaviors, help‐seeking intentions, and barriers. Table S2. Key characteristics for studies reporting help‐seeking behaviors. Table S3. Key characteristics for studies reporting help‐seeking intentions. Table S4. Prevalence rates of help‐seeking behaviors by time point and type of help. Table S5. Prevalence rates of help‐seeking intentions by type of help. Table S6. Risk of bias assessment of included studies. [file JCPP-66-1593-s001.docx]

# Supporting information

***Help-Seeking Intentions, Behavior, and Barriers in College Students: A Systematic Review and Meta-Analysis***

## *Ruiying Zhao, Yagmur Amanvermez, Julia Pei, Franchesca Castro-Ramirez, Charlene Rapsey, Claudia Garcia, David D. Ebert, Josep Maria Haro, Liviu A. Fodor, Oana A. David, Osiris Rankin, Sook Ning Chua, Vania Martínez, Ronny Bruffaert, Ronald C. Kessler, Pim Cuijpers*

Contents

[Supporting information 1](#_Toc183030628)

[*Ruiying Zhao, Yagmur Amanvermez, Julia Pei, Franchesca Castro-Ramirez, Charlene Rapsey, Claudia Garcia, David D. Ebert, Josep Maria Haro, Liviu A. Fodor, Oana A. David, Osiris Rankin, Sook Ning Chua, Vania Martínez, Ronny Bruffaert, Ronald C. Kessler, Pim Cuijpers* 1](#_Toc183030629)

[Appendix S1. Full search strings 2](#_Toc183030630)

[Appendix S2. The modified Newcastle-Ottawa Scale 3](#_Toc183030631)

[Table S1. References of included studies and their alignment with help-seeking behaviors, help-seeking intentions, and barriers 4](#_Toc183030632)

[Table S2. Key characteristics for studies reporting help-seeking behaviors 12](#_Toc183030633)

[Table S3. Key characteristics for studies reporting help-seeking intentions 17](#_Toc183030634)

[Figure S1. Funnel plot for prevalence rates of help-seeking behaviors among college students 19](#_Toc183030635)

[Figure S2. Funnel plot for prevalence rates of help-seeking intentions among college students 20](#_Toc183030636)

[Table S4. Prevalence rates of help-seeking behaviors by time point and type of help 21](#_Toc183030637)

[Table S5. Prevalence rates of help-seeking intentions by type of help 22](#_Toc183030638)

[Table S6. Risk of bias assessment of included studies 23](#_Toc183030639)

## Appendix S1. Full search strings

**PubMed**

(("Students"[Title/Abstract] OR "doctoral"[Title/Abstract] OR "PhD"[Title/Abstract]) AND ("university"[Title/Abstract] OR "college"[Title/Abstract] OR "higher education"[Title/Abstract]) AND ("help-seeking"[All Fields] OR ("uptake"[All Fields] OR "uptakes"[All Fields] OR "uptaking"[All Fields]) OR ("service"[All Fields] OR "service s"[All Fields] OR "serviced"[All Fields] OR "services"[All Fields] OR "services s"[All Fields] OR "servicing"[All Fields]) OR ("counsel"[All Fields] OR "counseled"[All Fields] OR "counselings"[All Fields] OR "counselled"[All Fields] OR "counselling"[All Fields] OR "counseling"[MeSH Terms] OR "counseling"[All Fields] OR "counsellings"[All Fields] OR "counsels"[All Fields]) OR ("psychotherapie"[All Fields] OR "psychotherapy"[MeSH Terms] OR "psychotherapy"[All Fields] OR "psychotherapies"[All Fields] OR "psychotherapy s"[All Fields]) OR ("therapeutics"[MeSH Terms] OR "therapeutics"[All Fields] OR "therapies"[All Fields] OR "therapy"[MeSH Subheading] OR "therapy"[All Fields] OR "therapy s"[All Fields] OR "therapys"[All Fields])) AND ("Mental Disorders"[MeSH Terms] OR "Substance-Related Disorders"[MeSH Terms])) NOT ("clinical trial"[Publication Type] OR "meta analysis"[Publication Type] OR "randomized controlled trial"[Publication Type] OR "systematic review"[Filter])

**Embase**

((students:ti,ab,kw OR doctoral:ti,ab,kw OR phd:ti,ab,kw) AND (university:ti,ab,kw OR college:ti,ab,kw) AND ('help seeking' OR uptake OR services OR psychotherapy OR counseling OR counselling OR therapy) AND 'mental disorders'/exp/mj) NOT ('controlled study'/de OR 'meta analysis'/de OR 'randomized controlled trial'/de OR 'systematic review'/de)

**PsycINFO**

S2 TI ( students OR doctoral OR phd ) OR AB ( students OR doctoral OR phd ) OR DE ( students OR doctoral OR phd ) OR (MA university or college or higher education)

S3 TI ( university or college or higher education ) OR AB ( university or college or higher education ) OR DE ( university or college or higher education )

S4 help-seeking OR uptake OR services OR psychotherapy OR counseling OR counselling OR therapy

S6 DE mental disorders or mental health or mental illness

s2 and s3 and s4 and s6

Limits: Methodology: - prospective study; - followup study; - longitudinal study; - quantitative study; - empirical study;

## Appendix S2. The modified Newcastle-Ottawa Scale

**(1) Sample representativeness:**

- 1 point: Population contained multiple university years at multiple universities.
- 0 points: Population contained either a single university year, a single university, or both.

**(2) Sample size:**

- 1 point: Sample size (the number of students who participated in the study) was greater than or equal to 1,500 participants.
- 0 points: Sample size (the number of students who participated in the study) was less than 1,500 participants.

**(3) Non-respondents:**

- 1 point: Comparability between respondent and non-respondent characteristics was established with a satisfactory response rate (i.e > 60%)
- 0 points: The response rate was unsatisfactory, the comparability between respondents and non-respondents was unsatisfactory, or there was no description of the response rate or the characteristics of the responders and the non-responders.

**(4) Ascertainment of help-seeking intention/ behaviors or reasons not to seek help in college students with mental health problems:**

- 1 point: A validated measurement tool (e.g. diagnostic interview or self-report scale) was used to identify the college students with a mental disorder.
- 0 points: No validated measurement tool for mental health assessment was described or referenced.

**(5) Quality of descriptive statistics reporting:**

- 1 point: Reported descriptive statistics to describe the population (e.g., age and gender distribution) with proper measures of dispersion (e.g., mean, standard deviation).
- 0 points: Descriptive statistics were not reported, or were incomplete, or did not report measures of dispersion.

## Table S1. References of included studies and their alignment with help-seeking behaviors, help-seeking intentions, and barriers

| **No** | **Full-citation** | **Help-seeking behaviors** | **Help-seeking intentions** | **Barriers** |
| --- | --- | --- | --- | --- |
| 1 | Adams, K. L., Saunders, K. E., Keown-Stoneman, C. D. G., & Duffy, A. C. (2021). Mental health trajectories in undergraduate students over the first year of university: a longitudinal cohort study. *BMJ Open*, *11*(12), e047393. | **x** |  |  |
| 2 | Amone-P’Olak, K., Kakinda, A. I., Kibedi, H., & Omech, B. (2023). Barriers to treatment and care for depression among the youth in Uganda: The role of mental health literacy. *Frontiers in Public Health*, *11*. |  |  | **x** |
| 3 | Arria, A. M., Winick, E. R., Garnier-Dykstra, L. M., Vincent, K. B., Caldeira, K. M., Wilcox, H. C., & O’Grady, K. E. (2011). Help Seeking and Mental Health Service Utilization Among College Students With a History of Suicide Ideation. *Psychiatric Services*, *62*(12), 1510–1513. | **x** |  |  |
| 4 | Baklola, M., Terra, M., Elzayat, M. A., Abdelhady, D., El-Gilany, A.-H., & collaborators, A. team of. (2023). Pattern, barriers, and predictors of mental health care utilization among Egyptian undergraduates: a cross-sectional multi-centre study. *BMC Psychiatry*, *23*(1), 139. | **x** |  | **x** |
| 5 | Benjet, C., Wittenborn, A., Gutierrez-García, R. A., Albor, Y. C., Contreras, E. V., Hernández, S. C., … Bruffaerts, R. (2020). Treatment Delivery Preferences Associated With Type of Mental Disorder and Perceived Treatment Barriers Among Mexican University Students. *Journal of Adolescent Health*, *67*(2), 232–238. |  | **x** |  |
| 6 | Bernhardsdóttir, J., & Vilhjálmsson, R. (2013). Psychological distress among university female students and their need for mental health services. *Journal of Psychiatric and Mental Health Nursing*, *20*(8), 672–678. | **x** | **x** |  |
| 7 | Bilican, F. I. (2013). Help-Seeking Attitudes and Behaviors Regarding Mental Health Among Turkish College Students. *International Journal of Mental Health*, *42*(2-3), 43–59. | **x** |  |  |
| 8 | Blanco, C., Okuda, M., Wright, C., Hasin, D. S., Grant, B. F., Liu, S.-M., & Olfson, M. (2008). Mental Health of College Students and Their Non–College-Attending Peers. *Archives of General Psychiatry*, *65*(12), 1429–1437. | **x** |  |  |
| 9 | Bootsma, E., Jansen, L., Kiekens, G., Voorpoels, W., Mortier, P., Proost, S., … Bruffaerts, R. (2023). Mood disorders in higher education in Flanders during the 2nd and 3 COVID-19 wave: Prevalence and help-seeking: Findings from the Flemish College Surveys (FLeCS). *Journal of Psychiatric Research*, *159*, 33–41. | **x** | **x** |  |
| 10 | Borsari, B., Yalch, M. M., Pedrelli, P., Radomski, S., Bachrach, R. L., & Read, J. P. (2018). Associations among trauma, depression, and alcohol use profiles and treatment motivation and engagement in college students. *Journal of American College Health*, *66*(7), 644–654. | **x** | **x** |  |
| 11 | Bovier, P. A., Chamot, E., Eytan, A., & Perneger, T. V. (2001). Patterns of Use of Ambulatory Mental Health Services in a Universal Care Setting. *Psychiatric Services*, *52*(11), 1515–1520. | **x** |  |  |
| 12 | Bruffaerts, R., Mortier, P., Auerbach, R. P., Alonso, J., Hermosillo De la Torre, A. E., Cuijpers, P., … Kessler, R. C. (2019). Lifetime and 12‐month treatment for mental disorders and suicidal thoughts and behaviors among first year college students. *International Journal of Methods in Psychiatric Research*, *28*(2), e1764. | **x** |  |  |
| 13 | Buscemi, J., Murphy, J. G., Martens, M. P., McDevitt-Murphy, M. E., Dennhardt, A. A., & Skidmore, J. R. (2010). Help-seeking for alcohol-related problems in college students: Correlates and preferred resources. *Psychology of Addictive Behaviors*, *24*(4), 571–580. | **x** | **x** |  |
| 14 | Caldeira, K. M., Kasperski, S. J., Sharma, E., Vincent, K. B., O’Grady, K. E., Wish, E. D., & Arria, A. M. (2009). College students rarely seek help despite serious substance use problems. *Journal of Substance Abuse Treatment*, *37*(4), 368–378. | **x** |  |  |
| 15 | Capron, D. W., Bauer, B. W., Madson, M. B., & Schmidt, N. B. (2017). Treatment Seeking among College Students with Comorbid Hazardous Drinking and Elevated Mood/Anxiety Symptoms. *Substance Use & Misuse*, *53*(6), 1041–1050. |  | **x** |  |
| 16 | Cranford, J. A., Eisenberg, D., & Serras, A. M. (2009). Substance use behaviors, mental health problems, and use of mental health services in a probability sample of college students. *Addictive Behaviors*, *34*(2), 134–145. | **x** | **x** |  |
| 17 | Currier, J. M., McDermott, R. C., & Sims, B. M. (2016). Patterns of help-seeking in a national sample of student veterans: a matched control group investigation. *General Hospital Psychiatry*, *43*, 58–62. | **x** |  |  |
| 18 | Czyz, E. K., Horwitz, A. G., Eisenberg, D., Kramer, A., & King, C. A. (2013). Self-reported Barriers to Professional Help Seeking Among College Students at Elevated Risk for Suicide. *Journal of American College Health*, *61*(7), 398–406. |  |  | **x** |
| 19 | Davis, D. A., & Widseth, J. C. (1977). Prediction of help-seeking with the MMPI: The problem of base rates. *Journal of Clinical Psychology*, *33*(4), 995–1000. | **x** |  |  |
| 20 | de Paula, W., Pereira, J. M., Guimarães, N. S., Godman, B., Nascimento, R. C. R. M. do, & Meireles, A. L. (2022). Key characteristics including sex, sexual orientation and internet use associated with worse mental health among university students in Brazil and implications. *Journal of Public Health*, *44*(4), e487–e498. | **x** |  |  |
| 21 | Dunbar, M. S., Sontag-Padilla, L., Kase, C. A., Seelam, R., & Stein, B. D. (2018). Unmet Mental Health Treatment Need and Attitudes Toward Online Mental Health Services Among Community College Students. *Psychiatric Services*, *69*(5), 597–600. | **x** | **x** |  |
| 22 | Dunbar, M. S., Sontag-Padilla, L., Ramchand, R., Seelam, R., & Stein, B. D. (2017). Mental Health Service Utilization Among Lesbian, Gay, Bisexual, and Questioning or Queer College Students. *Journal of Adolescent Health*, *61*(3), 294–301. | **x** |  |  |
| 23 | Ebert, D. D., Mortier, P., Kaehlke, F., Bruffaerts, R., Baumeister, H., Auerbach, R. P., … Kessler, R. C. (2019). Barriers of mental health treatment utilization among first‐year college students: First cross‐national results from the WHO World Mental Health International College Student Initiative. *International Journal of Methods in Psychiatric Research*, *28*(2), e1782. |  |  | **x** |
| 24 | Eisenberg, D., Golberstein, E., & Gollust, S. E. (2007). Help-Seeking and Access to Mental Health Care in a University Student Population. *Medical Care*, *45*(7), 594–601. | **x** | **x** | **x** |
| 25 | Eisenberg, D., Nicklett, E. J., Roeder, K., & Kirz, N. E. (2011). Eating Disorder Symptoms Among College Students: Prevalence, Persistence, Correlates, and Treatment-Seeking. *Journal of American College Health*, *59*(8), 700–707. | **x** | **x** |  |
| 26 | Encrenaz, G., & Messiah, A. (2006). Lifetime psychiatric comorbidity with substance use disorders: Does healthcare use modify the strength of associations ? *Social Psychiatry and Psychiatric Epidemiology*, *41*(5), 378–385. | **x** |  |  |
| 27 | Epler, A. J., Sher, K. J., Loomis, T. B., & O’Malley, S. S. (2009). College Student Receptiveness to Various Alcohol Treatment Options. *Journal of American College Health*, *58*(1), 26–32. |  | **x** |  |
| 28 | Gebreegziabher, Y., Girma, E., & Tesfaye, M. (2019). Help-seeking behavior of Jimma university students with common mental disorders: A cross-sectional study. *PLOS ONE*, *14*(2), e0212657. | **x** |  |  |
| 29 | Givens, J. L., & Tjia, J. (2002). Depressed Medical Studentsʼ Use of Mental Health Services and Barriers to Use. *Academic Medicine*, *77*(9), 918–921. | **x** |  | **x** |
| 30 | Healthy Minds Study - Student Survey (D. Eisenberg, personal communication, 2021). https://healthymindsnetwork.org/ | **x** | **x** |  |
| 31 | Horgan, A., Kelly, P., Goodwin, J., & Behan, L. (2018). Depressive Symptoms and Suicidal Ideation among Irish Undergraduate College Students. *Issues in Mental Health Nursing*, *39*(7), 575–584. | **x** |  |  |
| 32 | Horwitz, A. G., McGuire, T., Busby, D. R., Eisenberg, D., Zheng, K., Pistorello, J., … King, C. A. (2020). Sociodemographic differences in barriers to mental health care among college students at elevated suicide risk. *Journal of Affective Disorders*, *271*, 123–130. | **x** |  | **x** |
| 33 | Hubbard, K., Reohr, P., Tolcher, L., & Downs, A. (2018). Stress, Mental Health Symptoms, and Help-Seeking in College Students. *Psi Chi Journal of Psychological Research*, *23*(4), 293–305. | **x** |  |  |
| 34 | Janota, M., Kovess-Masfety, V., Gobin-Bourdet, C., & Husky, M. M. (2022). Use of mental health services and perceived barriers to access services among college students with suicidal ideation. *Journal of Behavioral and Cognitive Therapy*, *32*(3), 183–196. | **x** | **x** | **x** |
| 35 | Jennings, K. S., Cheung, J. H., Britt, T. W., Goguen, K. N., Jeffirs, S. M., Peasley, A. L., & Lee, A. C. (2015). How are perceived stigma, self-stigma, and self-reliance related to treatment-seeking? A three-path model. *Psychiatric Rehabilitation Journal*, *38*(2), 109–116. | **x** |  |  |
| 36 | Knipe, D., Maughan, C., Gilbert, J., Dymock, D., Moran, P., & Gunnell, D. (2018). Mental health in medical, dentistry and veterinary students: cross-sectional online survey. *BJPsych Open*, *4*(6), 441–446. | **x** |  | **x** |
| 37 | Levin, M. E., Krafft, J., & Levin, C. (2018). Does self-help increase rates of help seeking for student mental health problems by minimizing stigma as a barrier? *Journal of American College Health*, *66*(4), 302–309. | **x** | **x** |  |
| 38 | Lintvedt, O. K., Knud S⊘rensen, Ostvik, A., Bas Verplanken, & Arfwedson, E. (2008). The Need for Web-Based Cognitive Behavior Therapy Among University Students. *Journal of Technology in Human Services*, *26*(2-4), 239–258. | **x** |  |  |
| 39 | Lipson, S. K., Zhou, S., Abelson, S., Heinze, J., Jirsa, M., Morigney, J., … Eisenberg, D. (2022). Trends in college student mental health and help-seeking by race/ethnicity: Findings from the national healthy minds study, 2013–2021. *Journal of Affective Disorders*, *306*, 138–147. | **x** |  |  |
| 40 | Lipson, S. K., Kern, A., Eisenberg, D., & Breland-Noble, A. M. (2018). Mental Health Disparities Among College Students of Color. *Journal of Adolescent Health*, *63*(3), 348–356. |  |  | **x** |
| 41 | Lipson, S. K., Lattie, E. G., & Eisenberg, D. (2019). Increased Rates of Mental Health Service Utilization by U.S. College Students: 10-Year Population-Level Trends (2007–2017). *Psychiatric Services*, *70*(1), 60–63. | **x** |  |  |
| 42 | Lipson, S. K., Zhou, S., Wagner, B., Beck, K., & Eisenberg, D. (2016). Major Differences: Variations in Undergraduate and Graduate Student Mental Health and Treatment Utilization Across Academic Disciplines. *Journal of College Student Psychotherapy*, *30*(1), 23–41. | **x** |  |  |
| 43 | McMichael, A. J., & Hetzel, B. S. (1974). Patterns of help-seeking for mental illness among Australian University students: An epidemiological study. *Social Science & Medicine (1967)*, *8*(4), 197–206. | **x** |  |  |
| 44 | Nam, B., Wilcox, H. C., Hilimire, M., & DeVylder, J. E. (2018). Perceived need for care and mental health service utilization among college students with suicidal ideation. *Journal of American College Health*, *66*(8), 713–719. | **x** | **x** |  |
| 45 | Negash, A., Khan, M. A., Medhin, G., Wondimagegn, D., & Araya, M. (2020). Mental distress, perceived need, and barriers to receive professional mental health care among university students in Ethiopia. *BMC Psychiatry*, *20*(1), 187. | **x** | **x** | **x** |
| 46 | Ohayon, M. M., & Roberts, L. W. (2014). Links between occupational activities and depressive mood in young adult populations. *Journal of Psychiatric Research*, *49*, 10–17. | **x** |  |  |
| 47 | Pham Tien, N., Pham Thanh, T., Nguyen Hanh, D., Duong Hoang, A., Bui Dang The, A., Kim Bao, G., … Hoang Van, M. (2020). Utilization of mental health services among university students in Vietnam. *International Journal of Mental Health*, *50*(2), 113–135. | **x** |  |  |
| 48 | Rith-Najarian, L., Sun, W., Chen, A., Chorpita, B., Chavira, D., Mougalian, S., & Gong-Guy, E. (2019). What’s in a name? Branding of online mental health programming for university students. *Journal of Consulting and Clinical Psychology*, *87*(4), 380–339. | **x** |  |  |
| 49 | Robinson, A. M., Jubenville, T. M., Renny, K., & Cairns, S. L. (2016). Academic and mental health needs of students on a Canadian campus. *Canadian Journal of Counselling and Psychotherapy*, *50*(2). |  | **x** | **x** |
| 50 | Romano, K. A., Lipson, S. K., Beccia, A. L., Quatromoni, P. A., Gordon, A. R., & Murgueitio, J. (2022). Changes in the prevalence and sociodemographic correlates of eating disorder symptoms from 2013 to 2020 among a large national sample of U.S. young adults: A repeated cross‐sectional study. *International Journal of Eating Disorders*, *55*(6), 776–789. | **x** |  |  |
| 51 | Sasaki, M. (2007). Barriers to Use of Mental Health Services by Japanese University Students. *Psychological Reports*, *100*(2), 400–406. |  | **x** |  |
| 52 | Schweitzer, R., McLean, J., & Klayich, M. (1995). Suicidal Ideation and Behaviours among University Students in Australia. *Australian & New Zealand Journal of Psychiatry*, *29*(3), 473–479. | **x** |  |  |
| 53 | Seehuus, M., Moeller, R. W., & Peisch, V. (2019). Gender effects on mental health symptoms and treatment in college students. Journal of American College Health, 69(1), 1–8. | **x** |  |  |
| 54 | Sonneville, K. R., & Lipson, S. K. (2018). Disparities in eating disorder diagnosis and treatment according to weight status, race/ethnicity, socioeconomic background, and sex among college students. *International Journal of Eating Disorders*, *51*(6), 518–526. | **x** | **x** | **x** |
| 55 | Sontag-Padilla, L., Woodbridge, M. W., Mendelsohn, J., D’Amico, E. J., Osilla, K. C., Jaycox, L. H., … Stein, B. D. (2016). Factors Affecting Mental Health Service Utilization Among California Public College and University Students. *Psychiatric Services*, *67*(8), 890–897. | **x** |  |  |
| 56 | Valenstein, M., Clive, R., Ganoczy, D., Garlick, J., Walters, H. M., West, B. T., … Pfeiffer, P. N. (2022). A nationally representative sample of veteran and matched non-veteran college students: Mental health symptoms, suicidal ideation, and mental health treatment. *Journal of American College Health*, *70*(2), 436–445. | **x** | **x** |  |
| 57 | Verger, P., Guagliardo, V., Gilbert, F., Rouillon, F., & Kovess-Masfety, V. (2010). Psychiatric disorders in students in six French universities: 12-month prevalence, comorbidity, impairment and help-seeking. *Social Psychiatry and Psychiatric Epidemiology*, *45*(2), 189–199. | **x** |  |  |
| 58 | Wadman, R., Webster, L., Mawn, L., & Stain, H. J. (2019). Adult attachment, psychological distress and help-seeking in university students: Findings from a cross-sectional online survey in England. *Mental Health & Prevention*, *13*, 7–13. | **x** |  |  |
| 59 | Whitlock, J., Eckenrode, J., & Silverman, D. (2006). Self-injurious Behaviors in a College Population. *Pediatrics*, *117*(6), 1939–1948. | **x** |  |  |
| 60 | Whitlock, J., Muehlenkamp, J., Purington, A., Eckenrode, J., Barreira, P., Baral Abrams, G., … Knox, K. (2011). Nonsuicidal self-injury in a college population: general trends and sex differences. *Journal of American College Health*, *59*(8), 691–698. | **x** |  |  |
| 61 | Wu, L.-T., Pilowsky, D. J., Schlenger, W. E., & Hasin, D. (2007). Alcohol Use Disorders and the Use of Treatment Services Among College-Age Young Adults. *Psychiatric Services*, *58*(2), 192–200. | **x** | **x** | **x** |
| 62 | Xiao, P., Chen, L., Dong, X., Zhao, Z., Yu, J., Wang, D., & Li, W. (2022). Anxiety, Depression, and Satisfaction With Life Among College Students in China: Nine Months After Initiation of the Outbreak of COVID-19. *Frontiers in Psychiatry*, *12*. | **x** |  |  |

*Note.* An “**x**” indicates that the study addresses the respective aim (help-seeking behaviors, help-seeking intentions, or barriers).

## Table S2. Key characteristics for studies reporting help-seeking behaviors

| **Study** | **Country** | **Study Design** | **Type of Student** | **Age (M)** | **Female (%)** | **Recruit** | **Compen** | **MHC** | **HSB** | **HSB Time Frame** | ***n* HSB** | ***N* Total** |
| --- | --- | --- | --- | --- | --- | --- | --- | --- | --- | --- | --- | --- |
| Adams et al., 2021 | Canada | Longitudinal | Undergrad | 18.00 | 72 | General | Yes | Depression | Any formal help | Current | 46 | 450 |
| Arria et al., 2011 | USA | Longitudinal | Undergrad | N.S. | 66 | General | N.S. | Suicidal thoughts | Any formal help | Lifetime | 63 | 94 |
| Baklola et al., 2023 | Egypt | Cross-sectional | Undergrad | 20.68 | 65 | General | N.S. | Psychological distress | Any formal help | N.S. | 247 | 2207 |
| Bernhardsdóttir and Vilhjálmsson, 2013 | EU | Cross-sectional | Mixed | 27.90 | 100 | General | N.S. | Depression | Any formal help | Current | 47 | 163 |
| Bilican, 2013 | Turkey | Cross-sectional | Undergrad | 20.61 | 61 | General | Yes | Depression | Psychotherapy or counseling | Lifetime | 4 | 31 |
| Blanco et al., 2008 | USA | Cross-sectional | Mixed | N.S. | 53 | General | N.S. | Any axis-I disorder (past-year diagnosis of alcohol use disorder, drug use disorder, any mood disorder, or any anxiety disorder) | Any formal help | 12-month | 161 | 872 |
| Bootsma et al., 2023 | EU | Cross-sectional | Mixed | 20.20 | 56 | Mixed | No | Any mood disorders (major depressive episode, mania, hypomania) | Psychological counseling or medication | 12-month | 1477 | 4608 |
| Borsari et al., 2018 | USA | Cross-sectional | Undergrad | 19.29 | 54 | Mixed | Yes | Concomitant (defined based on depressive and PTSD symptoms) | Psychotherapy or counseling | Current | 1 | 35 |
| Bovier et al., 2001 | EU | Cross-sectional | Mixed | 26.00 | 58 | General | N.S. | Psychological distress | Any formal help | 12-month | 37 | 124 |
| Bruffaerts et al., 2019 | EU | Cross-sectional | Undergrad | 19.33 | 58 | General | Yes | Any mental disorder | Any formal help | 12-month | 1111 | 4391 |
| Buscemi et al., 2010 | USA | Cross-sectional | Undergrad | 19.42 | 54 | Mixed | N.S. | Depression | Any formal help | Lifetime | 3 | 55 |
| Caldeira et al., 2009 | USA | Longitudinal | Undergrad | N.S. | 54 | General | Yes | Any substance use disorder | Any formal help | During college | 10 | 548 |
| Cranford et al., 2009 | USA | Cross-sectional | Mixed | N.S. | 50 | General | Yes | Frequent binge drinking and any of these mental health problems | Any formal help | 12-month | 57 | 151 |
| Currier et al., 2016 | USA | Cross-sectional | Mixed | N.S. | 29 | General | N.S. | Depression | Psychotherapy or medication | 12-month | 253 | 752 |
| Davis and Widseth, 1977 | USA | Longitudinal | Undergrad | N.S. | 0 | General | N.S. | Depression and Psychasthenia | Psychotherapy or counseling | During college | 51 | 83 |
| de Paula et al., 2022 | Brazil | Longitudinal | Undergrad | N.S. | 58 | General | N.S. | Depression | Psychotherapy or counseling | Current | 75 | 151 |
| Dunbar et al., 2017 | USA | Cross-sectional | Mixed | N.S. | 54 | General | N.S. | Psychological distress | Any formal help | During college | 1656 | 6283 |
| Dunbar et al., 2018 | USA | Cross-sectional | Other | N.S. | 54 | General | N.S. | Psychological distress | Any formal help | During college | 436 | 1557 |
| Eisenberg et al., 2007 | USA | Cross-sectional | Mixed | N.S. | 48 | General | Yes | Major depression and no anxiety | Any formal help | 12-month | 40 | 112 |
| Eisenberg et al., 2011 | USA | Longitudinal | Mixed | N.S. | 52 | General | N.S. | Eating disorder | Any formal help | 12-month | 47 | 228 |
| Encrenaz and Messiah, 2006 | EU | Cross-sectional | Undergrad | N.S. | 70 | General | N.S. | One non-substance related psychiatric disorders | Any formal help | Lifetime | 27 | 70 |
| Gebreegziabher et al., 2019 | Ethiopia | Cross-sectional | Undergrad | 21.16 | 29 | General | N.S. | Psychological distress | Any formal help | 2-week | 97 | 444 |
| Givens and Tjia, 2002 | USA | Cross-sectional | Other | N.S. | 57 | General | N.S. | Depression | Any formal help | Current | 10 | 46 |
| HMS Dataset, 2021 | USA | Cross-sectional | Mixed | N.S. | 77 | N.S. | N.S. | Any mental health problem (depression, anxiety, NSSI or suicidal ideation) | Any formal help | Current | 24779 | 63960 |
| Horgan et al., 2018 | UK | Cross-sectional | Undergrad | N.S. | 81 | General | N.S. | Depression | Any formal help | Lifetime | 41 | 118 |
| Horwitz et al., 2020 | USA | Cross-sectional | Mixed | N.S. | 62 | General | Yes | Elevated suicide risk | Any formal help | Current | 1163 | 5772 |
| Hubbard et al., 2018 | USA | Cross-sectional | Mixed | 19.60 | 67 | General | Yes | Depression | Any formal help | Lifetime | 60 | 111 |
| Janota et al., 2022 | EU | Longitudinal | Undergrad | 18.76 | 73 | General | N.S. | Suicidality | Any formal help | Current | 76 | 627 |
| Jennings et al., 2015 | USA | Cross-sectional | Undergrad | N.S. | 75 | Subject pool | Yes | Depression or alcohol problem | Any formal help | 12-month | 65 | 95 |
| Knipe et al., 2018 | UK | Cross-sectional | Mixed | N.S. | 76 | General | N.S. | Depression | Any formal help | 2-week | 5 | 24 |
| Levin et al., 2018 | USA | Cross-sectional | N.S. | 21.07 | 65 | Subject pool | Yes | Psychological distress | Any formal help | Lifetime | 104 | 200 |
| Lintvedt et al., 2008 | EU | Cross-sectional | Mixed | 23.10 | 72 | General | N.S. | Depression | Any formal help | 12-month | 15 | 87 |
| Lipson et al., 2016 | USA | Cross-sectional | Mixed | N.S. | 56 | General | Yes | Any mental health problem (depression, anxiety, NSSI or suicidal ideation) | Any formal help | 12-month | 8508 | 21460 |
| Lipson et al., 2019 | USA | Cross-sectional | Mixed | N.S. | 57 | General | Yes | Depression | Any formal help | 12-month | 19147 | 41299 |
| Lipson et al., 2022 | USA | Cross-sectional | Mixed | N.S. | 57 | General | Yes | Depression or anxiety, eating disorder | Any formal help | 12-month | 90996 | 190070 |
| McMichael and Hetzel, 1974 | Australia or New Zealand | Longitudinal | Undergrad | N.S. | 45 | General | N.S. | Psychological distress | Any formal help | N.S. | 136 | 525 |
| Nam et al., 2018 | USA | Cross-sectional | Undergrad | N.S. | 68 | Subject pool | Yes | Suicidal thoughts | Any formal help | 12-month | 75 | 190 |
| Negash et al., 2020 | Ethiopia | Cross-sectional | Undergrad | 21.53 | 40 | General | N.S. | Psychological distress | Any formal help | 3-month | 14 | 339 |
| Ohayon and Roberts, 2014 | USA | Cross-sectional | Mixed | N.S. | 42 | General | N.S. | Depression-MDD | Any formal help | 12-month | 50 | 104 |
| Pham Tien et al., 2021 | Vietnam | Cross-sectional | Mixed | 20.51 | 74 | General | N.S. | Depression and/or anxiety | Any formal help | 12-month | 239 | 1915 |
| Rith-Najarian et al., 2019 | USA | Cross-sectional | Mixed | Undergrad: 20.7; Graduate:28.3 | 80 | General | Yes | Clinically significant symptoms (depression and/or anxiety) | Any formal help | During college | 145 | 395 |
| Romano et al., 2022 | USA | Cross-sectional | Mixed | 23.23 | 66 | General | Yes | Eating disorder symptoms | Psychotherapy or counseling | 12-month | 24465 | 68433 |
| Schweitzer et al., 1995 | Australia or New Zealand | Cross-sectional | Undergrad | 21.90 | 66 | General | N.S. | Suicidal thoughts | Any formal help | 12-month | 204 | 1040 |
| Seehuus et al., 2021 | USA | Cross-sectional | Undergrad | 19.94 | 57 | General | Yes | Depression | Any formal help | During college | 274 | 520 |
| Sonneville and Lipson, 2018 | USA | Cross-sectional | Mixed | N.S. | 85 | General | N.S. | Eating disorder | Psychotherapy or counseling | 12-month | 238 | 1747 |
| Sontag-Padilla et al., 2016 | USA | Cross-sectional | Mixed | N.S. | 64 | General | N.S. | Psychological distress | Any formal help | During college | 1264 | 6322 |
| Valenstein et al., 2022 | USA | Cross-sectional | Mixed | 32.00 | 26 | General | Yes | Depression, anxiety or PTSD, Suicidal ideation and behaviors | Any formal help | 12-month | 287 | 802 |
| Verger et al., 2010 | EU | Cross-sectional | Undergrad | 19.40 | 63 | General | No | Any psychiatric disorder | Any formal help | 12-month | 134 | 440 |
| Wadman et al., 2019 | UK | Cross-sectional | N.S. | 20.73 | Mild/moderate 81.9%; Severe 85.3% | General | Yes | Psychological distress-severe | Psychotherapy or counseling | Current | 31 | 102 |
| Whitlock et al., 2006 | USA | Cross-sectional | Mixed | N.S. | 56 | General | Ns | Self-injurious behaviors without suicidal intent | Psychotherapy or counseling | Lifetime | 241 | 490 |
| Whitlock et al., 2011 | USA | Cross-sectional | Mixed | 20.50 | 71 | General | Yes | Non-suicidal Self-injury | Psychotherapy or counseling | Lifetime | 941 | 1776 |
| Wu et al., 2007 | USA | Cross-sectional | N.S. | N.S. | N.S. | General | N.S. | Alcohol use disorder | Any formal help | 12-month | 43 | 1005 |
| Xiao et al., 2022 | China | Cross-sectional | Undergrad | 19.58 | 58 | General | N.S. | Depression | Psychotherapy or counseling | 1-month | 219 | 2345 |

*Note.* Compen: compensation; HSB: help-seeking behaviors; MHC: mental health condition(s); N.S.: not specified; N total: the number of students who have mental health problems; Recruit: recruitment; Undergrad: undergraduate.

## Table S3. Key characteristics for studies reporting help-seeking intentions

|  |  |  |  |  |  |  |  |  |  |  |  |  |
| --- | --- | --- | --- | --- | --- | --- | --- | --- | --- | --- | --- | --- |
| **Study** | **Country** | **Study Design** | **Type of Student** | **Age (M)** | **Female (%)** | **Recruit** | **Compen** | **MHC** | **HSI** | **HSI Time Frame** | ***n* HIS** | ***N* Total** |
| Benjet et al., 2020 | Mexico | Cross-sectional | Undergrad | 19.37 | 55 | General | N.S. | Combined | Likelihood of help seeking | During the school year | 1482 | 2157 |
| Bernhardsdóttir and Vilhjálmsson, 2013 | EU | Cross-sectional | Mixed | 27.90 | 100 | General | N.S. | Depression | Perceived need | Current | 110 | 163 |
| Bootsma et al., 2023 | EU | Cross-sectional | Mixed | 20.20 | 56 | Mixed | No | Any mood disorders (major depressive episode, mania, hypomania) | Perceived need | 12-month | 1656 | 4608 |
| Borsari et al., 2018 | USA | Cross-sectional | Undergrad | 19.29 | 54 | General and subject pool | Yes | Combined | Perceived need | N.S. | 1 | 35 |
| Buscemi et al., 2010 | USA | Cross-sectional | Undergrad | 19.42 | 54 | Mixed | N.S. | Depression | Likelihood of help seeking | N.S. | 29 | 55 |
| Capron et al., 2018 | USA | Cross-sectional | Undergrad | N.S. | N.S. | Subject pool | Yes | Alcohol problems | Interest in treatment | N.S. | 7 | 222 |
| Cranford et al., 2009 | USA | Cross-sectional | Mixed | N.S. | 50 | General | N.S. | Combined | Perceived need | 12-month | 101 | 151 |
| Dunbar et al., 2018 | USA | Cross-sectional | Other | N.S. | 54 | General | N.S. | Psychological distress | Interest in treatment | N.S. | 1183 | 1557 |
| Eisenberg et al., 2007 | USA | Cross-sectional | Mixed | N.S. | 48 | General | Yes | Depression | Perceived need | 12-month | 81 | 112 |
| Eisenberg et al., 2011 | USA | Longitudinal | Mixed | N.S. | 52 | General | N.S. | Eating disorder | Perceived need | N.S. | 109 | 228 |
| Epler et al., 2009 | USA | Longitudinal | Undergrad | 21.00 | 61 | General | N.S. | Alcohol problems | Interest in treatment | N.S. | 59 | 192 |
| HMS Dataset, 2021 | USA | Cross-sectional | Mixed | N.S. | 77 | N.S. | N.S. | Combined | Perceived need | N.S. | 55966 | 65594 |
| Janota et al., 2022 | EU | Longitudinal | Undergrad | 18.76 | 73 | General | N.S. | Suicidality | Perceived need | 12-month | 210 | 627 |
| Levin et al., 2018 | USA | Cross-sectional | N.S. | 21.07 | 65 | Subject pool | Yes | Psychological distress | Likelihood of help seeking | N.S. | 72 | 200 |
| Nam et al., 2018 | USA | Cross-sectional | Undergrad | N.S. | 68 | Subject pool | Yes | Suicidality | Perceived need | 12-month | 107 | 190 |
| Negash et al., 2020 | Ethiopia | Cross-sectional | Undergrad | 21.53 | 40 | General | N.S. | Psychological distress | Perceived need | Past 3 month | 239 | 339 |
| Robinson et al., 2016 | Canada | Cross-sectional | Mixed | 21.00 | 60 | General | N.S. | Psychological distress | Likelihood of help seeking | Within the next month | 21 | 165 |
| Sasaki, 2007 | Japan | Cross-sectional | Undergrad | 20.90 | 49 | General | N.S. | Depression | Likelihood of help seeking & Perceived need | Lifetime | 18 | 55 |
| Sonneville and Lipson, 2018 | USA | Cross-sectional | Mixed | N.S. | 85 | General | N.S. | Eating disorder | Perceived need | 12-month | 536 | 1747 |
| Valenstein et al., 2022 | USA | Cross-sectional | Mixed | 32.00 | 26 | General | Yes | Combined | Perceived need | 12-month | 441 | 802 |
| Wu et al., 2007 | USA | Cross-sectional | N.S. | N.S. | N.S. | General | N.S. | Alcohol problems | Perceived need | 12-month | 28 | 962 |

*Note.* Compen: compensation; HSI: help-seeking intentions; MHC: mental health condition(s); N.S.:not specified; N total: the number of students who have mental health problems; Recruit: recruitment; Undergrad: undergraduate.

## Figure S1. Funnel plot for prevalence rates of help-seeking behaviors among college students


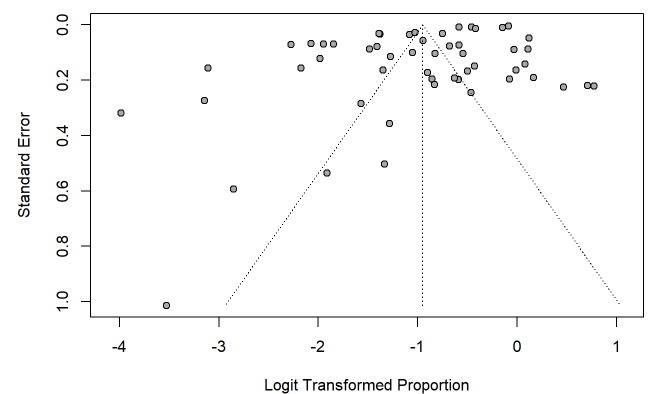


## Figure S2. Funnel plot for prevalence rates of help-seeking intentions among college students


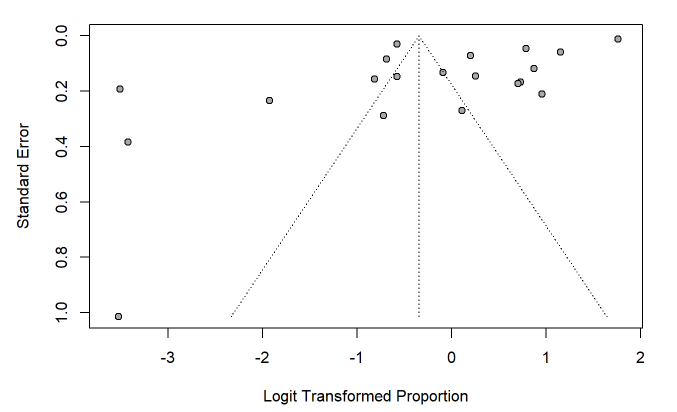


## Table S4. Prevalence rates of help-seeking behaviors by time point and type of help

|  |  |  |  |  |  |  |  |
| --- | --- | --- | --- | --- | --- | --- | --- |
|  | **No. of studies** | **No. HSB** | **Total No.** | **Prevalence (%)** | **95% CI** | ***I^2^* [95% CI]** | **Prediction interval** |
| **Help-seeking behaviors time point** | | |  |  |  |  |  |
| 12-month | 22 | 147649 | 339925 | 29 | 23–37 | 99.7% [99.6–99.7%] | 16-47 |
| Lifetime | 9 | 1484 | 2945 | 44 | 29–60 | 88.0% [79.3–93.0%] | 23–68 |
| Current | 9 | 26228 | 71306 | 23 | 13–37 | 99.2% [99.0–99.4%] | 5–62 |
| During college | 7 | 3836 | 15708 | 28 | 10–56 | 98.6% [98.1–99.0%] | 9–60 |
| Up to 3-month | 4 | 335 | 3152 | 12 | 3–35 | 95.9% [92.3–97.8%] | 0.4–81 |
|  |  |  |  |  |  |  |  |
| **Type of help** |  |  |  |  |  |  |  |
| Medication | 10 | 87507 | 280378 | 27 | 22–34 | 98.1% [97.4–98.6%] | 22–34 |
| Psychotherapy/counseling | 19 | 110502 | 309540 | 31 | 23–39 | 98.7% [98.5–98.9%] | 23–40 |

*Note.* HSB: help-seeking behaviors.

## Table S5. Prevalence rates of help-seeking intentions by type of help

|  |  |  |  |  |  |  |  |
| --- | --- | --- | --- | --- | --- | --- | --- |
|  | **No. of studies** | **No. HSI** | **Total No.** | **Prevalence (%)** | **95% CI** | ***I^2^* [95% CI]** | **Prediction interval** |
| **Type of intentions** |  |  |  |  |  |  |  |
| Perceived need only | 13 | 57949 | 70978 | 48 | 25–71 | 99.7% [99.7–99.8%] | 3–97 |
| Likelihood of seeking help | 5 | 1622 | 2632 | 39 | 15–69 | 98.2% [97.2–98.8%] | 1–97 |

*Note.* HSI: help-seeking intentions.

## Table S6. Risk of bias assessment of included studies

|  |  |  |  |  |  |  |  |
| --- | --- | --- | --- | --- | --- | --- | --- |
| **Study** | **Sample representativeness** | **Sample size** | **Non-respondents** | **Ascertainment of HSI/HSB** | **Descriptive statistics** | **Total score** | **RoB classification** |
| Adams et al., 2021 | 1 | 1 | 0 | 1 | 1 | 4 | Low risk |
| Amone-P'Olak et al., 2023 | 1 | 0 | 1 | 1 | 1 | 4 | Low risk |
| Arria et al., 2011 | 0 | 0 | 0 | 1 | 0 | 1 | High risk |
| Baklola et al., 2023 | 1 | 1 | 0 | 1 | 1 | 4 | Low risk |
| Benjet et al., 2020 | 1 | 1 | 0 | 1 | 1 | 4 | Low risk |
| Bernhardsdóttir and Vilhjálmsson, 2013 | 0 | 0 | 0 | 1 | 1 | 2 | High risk |
| Bilican, 2013 | 0 | 0 | 0 | 1 | 1 | 2 | High risk |
| Blanco et al., 2008 | 1 | 1 | 0 | 1 | 0 | 3 | Low risk |
| Bootsma et al., 2023 | 1 | 1 | 0 | 1 | 1 | 4 | Low risk |
| Borsari et al., 2018 | 0 | 0 | 0 | 1 | 1 | 2 | High risk |
| Bovier et al., 2001 | 0 | 0 | 0 | 1 | 1 | 2 | High risk |
| Bruffaerts et al., 2019 | 1 | 1 | 0 | 1 | 1 | 4 | Low risk |
| Buscemi et al., 2010 | 0 | 0 | 0 | 1 | 1 | 2 | High risk |
| Caldeira et al., 2009 | 0 | 0 | 1 | 1 | 0 | 2 | High risk |
| Capron et al., 2018 | 0 | 0 | 0 | 1 | 0 | 1 | High risk |
| Cranford et al., 2009 | 0 | 1 | 0 | 1 | 0 | 2 | High risk |
| Currier et al., 2016 | 1 | 1 | 0 | 1 | 1 | 4 | Low risk |
| Czyz et al., 2013 | 0 | 0 | 0 | 1 | 1 | 2 | High risk |
| Davis and Widseth, 1977 | 0 | 0 | 1 | 1 | 0 | 2 | High risk |
| de Paula et al., 2022 | 0 | 0 | 0 | 1 | 1 | 2 | High risk |
| Dunbar et al., 2017 | 1 | 1 | 0 | 1 | 0 | 3 | Low risk |
| Dunbar et al., 2018 | 1 | 1 | 0 | 1 | 0 | 3 | Low risk |
| Ebert et al., 2019 | 1 | 1 | 0 | 1 | 0 | 3 | Low risk |
| Eisenberg et al., 2007 | 0 | 1 | 0 | 1 | 0 | 2 | High risk |
| Eisenberg et al., 2011 | 0 | 1 | 0 | 1 | 1 | 3 | Low risk |
| Encrenaz and Messiah, 2006 | 0 | 1 | 0 | 1 | 1 | 3 | Low risk |
| Epler et al., 2009 | 0 | 1 | 0 | 1 | 1 | 3 | Low risk |
| Gebreegziabher et al., 2019 | 0 | 0 | 1 | 1 | 1 | 3 | Low risk |
| Givens and Tjia, 2002 | 0 | 0 | 1 | 1 | 0 | 2 | High risk |
| HMS Dataset, 2021 | N.S. | N.S. | N.S. | N.S. | N.S. | 0 | N.S. |
| Horgan et al., 2018 | 0 | 0 | 0 | 1 | 1 | 2 | High risk |
| Horwitz et al., 2020 | 1 | 1 | 0 | 1 | 1 | 4 | Low risk |
| Hubbard et al., 2018 | 1 | 0 | 0 | 1 | 1 | 3 | Low risk |
| Janota et al., 2022 | 0 | 0 | 0 | 1 | 1 | 2 | High risk |
| Jennings et al., 2015 | 0 | 0 | 0 | 1 | 1 | 2 | High risk |
| Knipe et al., 2018 | 0 | 0 | 0 | 1 | 1 | 2 | High risk |
| Levin et al., 2018 | 0 | 0 | 0 | 1 | 1 | 2 | High risk |
| Lintvedt et al., 2008 | 1 | 0 | 0 | 1 | 1 | 3 | Low risk |
| Lipson et al., 2016 | 1 | 1 | 0 | 1 | 0 | 3 | Low risk |
| Lipson et al., 2018 | 1 | 1 | 0 | 1 | 1 | 4 | Low risk |
| Lipson et al., 2019 | 1 | 1 | 0 | 1 | 0 | 3 | Low risk |
| Lipson et al., 2022 | 1 | 1 | 0 | 1 | 1 | 4 | Low risk |
| McMichael and Hetzel, 1974 | 0 | 1 | 1 | 1 | 0 | 3 | Low risk |
| Nam et al., 2018 | 0 | 0 | 0 | 1 | 0 | 1 | High risk |
| Negash et al., 2020 | 0 | 0 | 0 | 1 | 1 | 2 | High risk |
| Ohayon and Roberts, 2014 | 1 | 1 | 0 | 1 | 0 | 3 | Low risk |
| Pham Tien et al., 2021 | 1 | 1 | 0 | 1 | 1 | 4 | Low risk |
| Rith-Najarian et al., 2019 | 0 | 0 | 0 | 1 | 1 | 2 | High risk |
| Robinson et al., 2016 | 0 | 0 | 0 | 1 | 0 | 1 | High risk |
| Romano et al., 2022 | 1 | 1 | 0 | 1 | 1 | 4 | Low risk |
| Sasaki, 2007 | 1 | 0 | 0 | 1 | 1 | 3 | Low risk |
| Schweitzer et al., 1995 | 0 | 1 | 0 | 1 | 0 | 2 | High risk |
| Seehuus et al., 2021 | 1 | 1 | 0 | 1 | 1 | 4 | Low risk |
| Sonneville and Lipson, 2018 | 1 | 1 | 0 | 1 | 1 | 4 | Low risk |
| Sontag-Padilla et al., 2016 | 1 | 1 | 0 | 1 | 1 | 4 | Low risk |
| Valenstein et al., 2022 | 1 | 1 | 0 | 1 | 1 | 4 | Low risk |
| Verger et al., 2010 | 1 | 1 | 0 | 1 | 1 | 4 | Low risk |
| Wadman et al., 2019 | 0 | 0 | 0 | 1 | 1 | 2 | High risk |
| Whitlock et al., 2006 | 1 | 1 | 0 | 1 | 1 | 4 | Low risk |
| Whitlock et al., 2011 | 1 | 1 | 0 | 1 | 1 | 4 | Low risk |
| Wu et al., 2007 | 1 | 1 | 1 | 1 | 0 | 4 | Low risk |
| Xiao et al., 2022 | 1 | 1 | 0 | 1 | 1 | 4 | Low risk |

*Note.* HSI/HSB: help-seeking intention/help-seeking behaviors; N.S.: not specified; RoB: risk of bias.
